# Depletion of Hepatic SREBP2 Protects Against Hypercholesterolemia and Atherosclerosis through the ANGPTL3‐LPL Axis

**DOI:** 10.1002/advs.202412677

**Published:** 2025-03-19

**Authors:** Yifan Wang, Jia You Sarafina Choe, Yu Shi, Thi Tun Thi, Xiaoyun Cao, Yang Hu, Kai Yan Cheng, Hui Li, Yang Ji, Yan Liu, Matthew Ackers‐Johnson, Roger S.Y. Foo, Yujia Shen, Haojie Yu

**Affiliations:** ^1^ Department of Biochemistry Yong Loo Lin School of Medicine National University of Singapore Singapore 117596 Singapore; ^2^ Precision Medicine Translational Research Programme Yong Loo Lin School of Medicine National University of Singapore Singapore 117596 Singapore; ^3^ Cardiovascular Metabolic Disease Translational Research Programme Yong Loo Lin School of Medicine National University of Singapore Singapore 117599 Singapore; ^4^ Institute of Molecular and Cell Biology A*STAR Singapore 138673 Singapore; ^5^ Department of Medicine Yong Loo Lin School of Medicine National University of Singapore Singapore 117599 Singapore

**Keywords:** ANGPTL3, atherosclerosis, HoFH, LPL, SREBP2

## Abstract

Lipolysis of triglyceride‐rich lipoproteins by peripheral lipoprotein lipase (LPL) plays an essential role in maintaining systemic cholesterol/lipid homeostasis. Human genetic studies have unequivocally demonstrated that activation of LPL pathway reduces risks for both coronary artery disease (CAD) and type 2 diabetes (T2D). Although sterol regulatory element‐binding protein 2 (SREBP2) is well established as the master transcription factor that regulates the hepatic biosynthesis of both cholesterol and fatty acids, whether and how its activity in liver interacts with peripheral LPL pathway remains unknown. Here, it is demonstrated that acute liver‐specific depletion of SREBP2 results in divergent effects on the regulation of peripheral LPL activity in mice, depending on the presence or absence of low‐density lipoprotein receptors (LDLR). SREBP2 deficiency drastically elevates peripheral LPL activity through downregulation of plasma angiopoietin‐related protein 3 (ANGPTL3) levels in LDLR‐deficient mice. Moreover, in addition to SREBP2's transcriptional regulation of ANGPTL3, it is found that SREBP2 promotes proteasome‐based degradation of ANGPTL3 in the presence of LDLR. Remarkably, acute depletion of hepatic SREBP2 protects against hypercholesterolemia and atherosclerosis, in which atherosclerotic lesions are reduced by 45% compared to control littermates. Taken together, these findings outline a liver‐peripheral crosstalk mediated by SREBP2‐ANGPTL3‐LPL axis and suggest that SREBP2 inhibition can be an effective strategy to tackle homozygous familial hypercholesterolemia (HoFH).

## Graphical abstract

This study reveals a novel crosstalk between hepatic SREBP2‐mediated cholesterol biosynthesis pathway and peripheral lipoprotein lipase‐mediated lipolysis pathway in the regulation of systemic lipid metabolism. Notably, the lipid‐lowering effects of SREBP2 inhibition are independent of a functional LDL receptor, highlighting the potential of acute hepatic SREBP2 depletion as a promising therapeutic strategy for managing homozygous familial hypercholesterolemia.

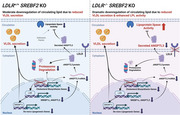

## 1 Introduction

Systemic cholesterol and lipid metabolism is tightly regulated through the interplay and cross‐communication of numerous pathways across a range of tissues and organs.^[^ ^1^ ^]^ Among these, the hepatic SREBP2 (sterol regulatory element‐binding protein 2)‐mediated cholesterol/fatty acid biosynthesis and peripheral LPL (lipoprotein lipase)‐mediated lipolysis of triglyceride (TG)‐rich lipoproteins are two major pathways that govern systemic cholesterol/lipid homeostasis.^[^ ^2^, ^3^ ^]^ Dysregulation of these pathways is associated with a variety of cardiovascular and metabolic diseases.^[^ ^4^, ^5^ ^]^

Lipoprotein lipase catalyzes the hydrolysis of intravascular TG packed in TG‐rich lipoproteins such as chylomicrons and very low‐density lipoproteins (VLDLs), providing free fatty acids for peripheral tissues. LPL is highly expressed in various tissues, including adipose tissue, skeletal and cardiac muscle, and macrophages. Its activity is subject to tissue‐specific regulation at different levels.^[^ ^3^ ^]^ Among these regulatory factors, liver‐derived circulating angiopoietin‐like protein 3 (ANGPTL3) plays a crucial role in modulating LPL activities.^[^ ^6^, ^7^, ^8^ ^]^ ANGPTL3 is exclusively expressed in the liver, and individuals with loss‐of‐function variants of ANGPTL3 are associated with combined hypolipidemia.^[^ ^7^ ^]^ In patients with homozygous familial hypercholesterolemia (HoFH), there is only a modest reduction in LDL‐cholesterol levels (typically in the range of 10–25%) even with the highest doses of the most potent statins,^[^ ^5^ ^]^ primarily due to the defect or complete loss of LDLR activity. However, the anti‐ANGPTL3 antibody Evinacumab has demonstrated promising lipid‐lowering effects that are independent of LDLR functionality and been approved for the treatment of HoFH. Clinical trials have shown that Evinacumab reduced LDL cholesterol by an average of 47% and triglycerides by 55% in patients with HoFH.^[^ ^9^, ^10^ ^]^ The expression of *ANGPTL3* in the liver is regulated at multiple levels, with reports suggesting that *ANGPTL3* is transcriptionally regulated by liver X receptor (LXR).^[^ ^11^ ^]^ This may partially explain why mice treated with an LXR agonist exhibit increased plasma cholesterol and TG levels.^[^ ^11^ ^]^

Sterol regulatory element‐binding protein 2, encoded by *SREBF2*, is the master transcription factor for cholesterol/fatty acid synthesis, and its activity is subject to feedback regulation by LDLR‐mediated cholesterol uptake.^[^ ^2^, ^12^ ^]^ Transgenic expression of mature SREBP2 in liver preferentially promotes expression of genes in the cholesterol biosynthetic pathway, such as *HMGCR*, *MVK*, as well as *LDLR* and *PCSK9*.^[^ ^13^ ^]^ Homozygous disruption of *Srebf2* in mice resulted in embryonic lethality.^[^ ^2^, ^14^ ^]^ However, mice with hepatocyte‐specific deletion of *Srebf2* are viable and have substantially reduced cholesterol and triglyceride (TG) contents in both liver and blood without affecting liver LDLR protein levels.^[^ ^15^ ^]^ Consistently, hepatic expression of both cholesterol biosynthetic genes and fatty acid biosynthetic genes downstream of SREBP‐1c are significantly downregulated in the absence of SREBP2.^[^ ^14^, ^15^ ^]^ Additionally, it's found that SREBP2‐mediated production of sterol ligand is required for LXR activation in the liver.^[^ ^15^ ^]^ Despite these findings, it remains unclear whether SREBP2 regulates ANGPTL3 and whether there is any crosstalk between SREBP2‐mediated lipid production and peripheral LPL‐mediated fatty acid uptake. Furthermore, it remains unknown whether the lipid‐lowering effects observed in *Srebf2* liver‐specific knockout mice are due to developmental defects or whether they depend on the presence of intact LDLR.

To address those questions, we employed a variety of mouse models in which the function of SREBP2 in the regulation of plasma lipid levels and peripheral LPL activity were studied. Acute depletion of hepatic SREBP2 via AAV‐shRNA or liver‐specific expression of Cre recombinase (AAV‐TBG‐Cre) reduced plasma triglyceride (TG) and total cholesterol (TC) levels in both *Ldlr^+/+^* and *Ldlr^−/−^* mice, suggesting that the lipid‐lowering effect upon SREBP2 depletion does not require LDLR activity. In line with this finding, we further demonstrated that acute depletion of hepatic SREBP2 exhibited a synergistic cholesterol lowering effect with proprotein convertase subtilisin kexin 9 (PCSK9) inhibition in *Ldlr^+/+^* mice. Remarkably, a single administration of AAV significantly attenuated atherosclerotic plaque progression in *Ldlr^−/−^* mice. Mechanistically, our findings demonstrate that SREBP2 regulates ANGPTL3 levels through both transcriptional and post‐translational mechanisms. While SREBP2 inhibition reduced VLDL secretion in both *Ldlr^+/+^* and *Ldlr^−/−^* mice, it specifically enhanced peripheral LPL activity in *Ldlr^−/−^* mice by reducing ANGPTL3 levels in the bloodstream. In summary, our data reveal a novel regulatory pathway of peripheral LPL activity via hepatic SREBP2 and indicate that targeting hepatic SREBP2 might be an efficient strategy for the treatment of HoFH.

## 2 Results

### 2.1 Acute Depletion of Hepatic SREBP2 Lowers Plasma Triglyceride and Cholesterol Levels in Adult Mice

To examine whether acute depletion of hepatic SREBP2 in adult mice would reduce plasma TG and total cholesterol (TC) levels, C57BL/6J mice or LDLR‐deficient *(Ldlr^−/−^)* mice were injected with AAV8‐shRNA to knock down the expression of *Srebf2* in the liver. The mRNA levels of *Srebf2* in liver were substantially reduced by ∼67% compared to scramble control at 3 weeks post injection (Figure S1A, Supporting Information). Even at 10 weeks post injection, *Srebf2* mRNA levels were still significantly reduced by ≈45% and ≈49% in C57BL/6J and *Ldlr^−/−^* mice, respectively, compared to scramble control (Figure S1B,C, Supporting Information).

**Figure 1:**
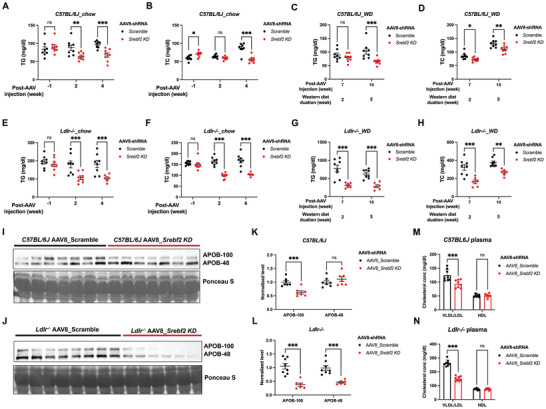
Acute depletion of hepatic SREBP2 reduces circulating lipid levels. A–D) Plasma triglyceride (A and C) and cholesterol (B and D) levels of 16 h fasted C57BL/6J male mice injected with AAV8‐scramble control or AAV8‐*Srebf2*‐shRNA viruses under 5‐week chow diet feeding (A and B, *n* = 8 mice per group, by student's *t* test) and 5‐week western diet feeding (C and D, *n* = 8 mice per group, by two‐way ANOVA). E–H) Plasma triglyceride (E and G) and cholesterol (F and H) levels of 16 h fasted male mice lacking LDLR injected with AAV8‐scramble control or AAV8‐*Srebf2*‐shRNA viruses under 5‐week chow diet (E and F, *n* = 8 mice per group, by student's *t* test) and 5‐week western diet (G and H, *n* = 6–8 mice per group, by two‐way ANOVA) feeding. I–L) Western blot (I and J) and relative quantification (K and L) of APOB‐100 and APOB‐48 in the plasma of 4 h fasted male C57BL/6J mice (I and K, *n* = 7 mice per group, by student's *t* test) and mice lacking LDLR (J and L, *n* = 6–8 mice per group, by student's *t* test) after AAV8 injection for 3 weeks. M,N) Quantification of plasma VLDL/LDL and HDL cholesterol levels in 16 h‐fasted C57BL/6J and *Ldlr^−/−^* mice injected with AAV8‐scramble control or AAV8‐*Srebf2*‐shRNA viruses. Mice were maintained on a 5‐week chow diet, followed by a 5‐week western diet after AAV injection (*n* = 6–8 mice per group, by student's *t* test). *p < 0.05, **p < 0.01, and ***p < 0.001; Error bar indicate mean ± SD.

Consistent with the lipid‐lowering effects observed in liver‐specific *Srebf2* knockout mice,^[^ ^15^ ^]^ plasma TG and TC levels were reduced by 31% and 37%, respectively, in chow‐fed C57BL/6J mice treated with *Srebf2*‐shRNA compared to scramble‐shRNA at 4 weeks post injection (**Figure** **1**A,B). Upon western diet (WD) feeding for 5 weeks, plasma TG and TC levels were reduced by 37% and 14%, respectively, in mice upon SREBP2 depletion compared to control littermates (Figure 1C,D). Of note, the lipid‐lowering effects in C57BL/6J mice last at least for 10 weeks post injection (Figure 1C,D).

Remarkably, SREBP2 depletion in chow‐fed *Ldlr^−/−^* mice led to 43% and 39% reduction in plasma TG and TC levels, respectively, compared to control littermates (Figure 1E,F). After western diet (WD) for 2 weeks, plasma TG and TC levels were reduced by 60% and 49%, respectively, in knockdown mice compared to control littermates (Figure 1G,H). Furthermore, the lipid‐lowering effects in *Ldlr^−/−^* mice last at least for 15 weeks post injection. In summary, these data indicate that the lipid‐lowering effect upon hepatic SREBP2 depletion does not rely on LDLR‐mediated LDL‐C uptake.

In line with reduced plasma TG and TC levels, circulating APOB levels were lower in knockdown mice compared to control littermates (Figure 1I–L). However, in *Ldlr^+/+^* mice (C57BL/6J), only APOB100 was significantly reduced in SREBP2‐deficent mice, whereas in *Ldlr^−/−^* mice, both APOB100 and APOB48 were substantially decreased in SREBP2‐deficient mice compared to their control littermates (Figure 1I–L). Consistent with the TG‐lowering effects and the reduction in plasma APOB levels, hepatic SREBP2 depletion significantly decreases plasma VLDL/LDL cholesterol levels, but not high‐density lipoprotein (HDL) cholesterol levels, regardless of LDLR deficiency (Figure 1M,N). It is worth noting that depletion of SREBP2 led to significantly reduced body weight in both C57BL/6J mice and LDLR‐deficient mice fed WD diet compared to control littermates (Figure S1D,E, Supporting Information), suggesting that hepatic SREBP2 also plays a role in regulating whole body energy homeostasis.

Importantly, the depletion of hepatic SREBP2 via AAV8‐shRNA did not lead to any detectable adverse effects during the study period. Furthermore, mRNA levels of genes encoding proinflammatory cytokines or liver injury markers remained unaffected following SREBP2 depletion (Figure S1F–I, Supporting Information). These findings indicate that acute inhibition of SREBP2 is likely an effective strategy for lowering plasma TC and TG levels.

### 2.2 Acute Depletion of SREBP2 Reduces Hepatic VLDL Secretion Rate through the Downregulation of Hepatic Cholesterol and Fatty Acid Biosynthesis

**Figure 2:**
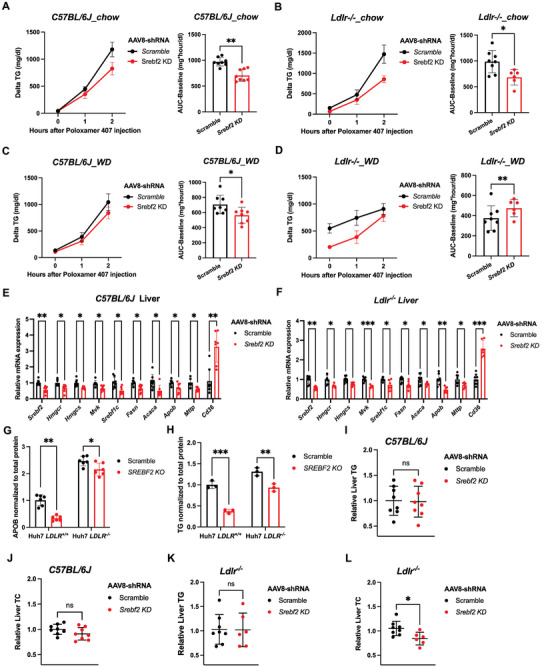
Knocking down hepatic *SREBF2* reduced VLDL secretion both in vitro and in vivo. A–D) Changes of plasma triglyceride after Poloxamer‐407 injection in male C57BL/6J mice (A and C) and male *Ldlr^−/−^* (B and D) injected with AAV8‐scramble control or AAV8‐*Srebf2*‐shRNA viruses under chow or western diet feeding. VLDL secretion levels were estimated and compared using an area‐under‐the‐curve (AUC) approach that subtracts the baseline value (*n* = 6–8 mice per group, by student's *t*‐test). E,F) Quantitative polymerase chain reaction (qPCR) expression analysis of *de novo* lipogenesis and cholesterol synthesis genes in liver of 16h‐fasted male C57BL/6J mice (E) and *Ldlr^−/−^* mice (F) injected with AAV8‐scramble control or AAV8‐*Srebf2*‐shRNA viruses under 5‐week chow followed by 5‐week western diet feeding (*n* = 6–8 per group, by student's *t* test). G) Measurement of APOB100 secretion levels in *SREBF2* knockout or control Huh7 cells in the presence or absence of LDLR using ELISA (*n* = 6 replicates per experiment, representative of 3 independent experiments, by two‐way ANOVA). H) Measurement of triglyceride secretion in the culture medium of *SREBF2* knockout or control Huh7 cells in the presence or absence of LDLR (*n* = 3 replicates per experiment, representative of 3 independent experiments, by two‐way ANOVA). I,K) Hepatic triglyceride contents in 16h‐fasted C57BL/6J (I) and *Ldlr^−/−^* (K) mice injected with AAV8‐scramble control or AAV8‐*Srebf2*‐shRNA viruses under 5‐week chow followed by 5‐week western diet feeding (*n* = 6–8 mice per group, by student's *t* test). J,L) Hepatic total cholesterol contents in 16h‐fasted C57BL/6J (J) and *Ldlr^−/−^* (L) mice injected with AAV8‐scramble control or AAV8‐*Srebf2*‐shRNA viruses under 5‐week chow followed by 5‐week western diet feeding (*n* = 6–8 mice per group, by student's *t* test). *p < 0.05, **p < 0.01, and ***p < 0.001; Error bars indicate mean ± SD.

Liver‐specific knockout of *Srebf2* in *Ldlr^+/+^* mice by Alb‐Cre resulted in substantially reduced plasma TG and TC levels, which is likely attributed to diminished hepatic VLDL secretion rate as hepatic LDLR protein levels are not impaired.^[^ ^15^ ^]^ To measure hepatic VLDL secretion, poloxamer‐407, an effective inhibitor of peripheral lipoprotein lipase, was administered in mice at 3 weeks (chow) or 8 weeks (3 weeks of WD) post AAV injection, and plasma TG and TC levels were monitored for 2h. Consistently, acute depletion of hepatic SREBP2 significantly reduced VLDL secretion in both C57BL/6J and LDLR‐deficient mice (3 weeks post injection) compared with control littermates fed chow (**Figure** **2**A,B). After western diet feeding for 3 weeks, knockdown of *Srebf2* only in C57BL/6J mice, rather than in LDLR‐deficient mice, led to a significantly decreased VLDL secretion rate (Figure 2C,D), suggesting that other lipid‐lowering pathways controlled by SREBP2 are involved in mice lacking LDLR upon WD feeding.

Quantitative real‐time PCR (qRT‐PCR) revealed that mRNA expression levels of SREBP2 downstream genes involved in cholesterol biosynthesis, such as *Hmgcr*, *Hmgcs* and *Mvk*, were all significantly reduced in both C57BL/6J and *Ldlr^−/−^* mice upon depletion of SREBP2 compared with control littermates (Figure 2E,F). Moreover, depletion of hepatic SREBP2 also attenuated the transcriptional activation of LXR and fatty acid biosynthesis genes, such as *Srebf1c*, *Fasn* and *Acaca*, in mice with or without LDLR (Figure 2E,F). Furthermore, mRNA levels of *Mttp* and *Apob* which play essential roles in VLDL assembly in hepatocytes^[^ ^16^, ^17^ ^]^ were also significantly reduced following SREBP2 depletion, suggesting a close interconnection between hepatic lipid production and the expression of VLDL packaging proteins, both of which fall under the transcriptional regulation of SREBP2 (Figure 2E,F). As compensation for reduced lipid production, hepatic expression of *Cd36*, which encodes free fatty acids transporter, is significantly upregulated in the absence of SREBP2 (Figure 2E,F). Therefore, the reduced hepatic VLDL secretion upon SREBP2 depletion is likely due to the downregulation of major pathways responsible for cholesterol/fatty acid biosynthesis and VLDL assembly.

To further investigate if SREBP2 directly regulates VLDL secretion in hepatocytes, we used CRISPR/Cas9 to knock out *SREBF2* in human hepatoma Huh7 cells and mouse hepatocyte AML12 cells with or without LDLR (Figure S2A–D, Supporting Information). In both *LDLR^+/+^* and *LDLR^−/−^* cells, CRISPR/Cas9‐mediated knockout reduced *SREBF2* mRNA by 60%–70% compared to scramble control (Figure S2F,G, Supporting Information). LDLR deficiency activated SREBP2 signaling and significantly increased SREBP2 precursor protein levels in Huh7 cells (Figure S2B,D, Supporting Information). Moreover, *SREBF2* itself, as well as its downstream target genes *HMGCR, HMGCS1, and MVK*, were significantly upregulated in the absence of LDLR in both mouse liver and in vitro‐cultured hepatocytes (Figure S2E–G, Supporting Information). In line with the in vivo findings, expression of genes related to VLDL assembly, cholesterol/ fatty acid biosynthesis, such as *HMGCR, FASN*, *SREBF1C, APOB*, and *MTTP*, were all significantly downregulated upon *SREBF2* knockout (Figure S2F,G, Supporting Information). We next employed an ELISA‐based assay to measure secreted levels of APOB100 in Huh7 cells and found that knockout of *SREBF2* in both *LDLR^+/+^* and *LDLR^−/−^* led to significantly reduced APOB100 levels in the medium compared to control cells with or without lipid mixture treatment (Figure 2G; Figure S2H, Supporting Information). Secreted TG levels in the culture medium were also diminished upon SREBP2 depletion (Figure 2H; Figure S2I, Supporting Information). Moreover, depletion of SREBP2 in *LDLR^+/+^* Huh7 cells has no effect on uptake of LDL particles, whereas in *LDLR^−/−^* cells, it leads to even slightly increased LDL uptake compared to control cells (Figure S2J, Supporting Information). Taken together, these data suggested that reduced VLDL production likely contributes to the diminished plasma TG and TC levels in SREBP2‐deficient mice.

Inhibition of hepatic VLDL production via cholesterol‐lowering therapies Lomitapide (MTP inhibitor) and Mipomersen (APOB antisense oligonucleotides) were accompanied by increased liver fat contents.^[^ ^18^ ^]^ To test if diminished VLDL production in *Srebf2*‐knockdown mice would also cause excessive lipid accumulation, we examined the liver TG/TC contents in both mouse strains. In both *Ldlr^+/+^* and *Ldlr^−/−^* mice fed WD for 5 weeks (10 weeks post injection), liver TG contents remain unchanged in *Srebf2* knockdown mice compared to control littermates (Figure 2I,K; Figure S3, Supporting Information). In contrast, liver TC contents were moderately reduced in SREBP2‐deficient mice compared to control littermates (Figure 2J,L; Figure S3, Supporting Information). Consistent with in vivo findings, knockout of *SREBF2* in Huh7 cells had no impact on cellular TG levels but did lead to a significant reduction in cholesterol levels compared to control cells (Figure S2K–N, Supporting Information). These data suggest that in hepatocytes lacking SREBP2, hepatic TG/TC contents arewell balanced through a combination of VLDL secretion and lipid biosynthesis.

### 2.3 Differential Regulatory Roles of SREBP2 on ANGPTL3 and Peripheral LPL Activity in the Presence or Absence of LDLR

**Figure 3:**
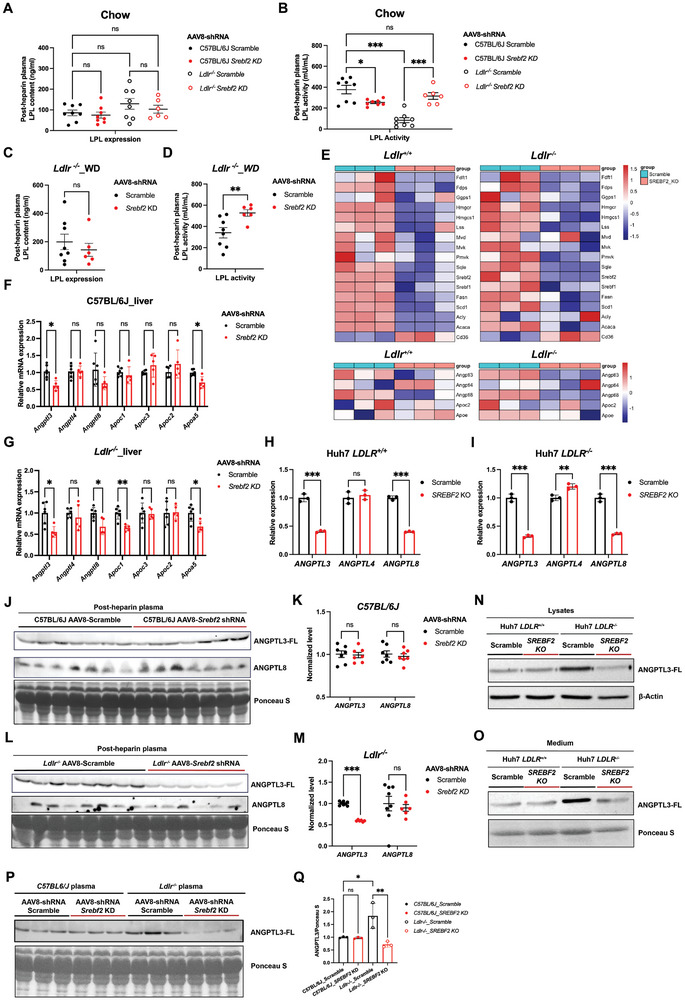
Depletion of hepatic SREBP2 promotes Lipoprotein lipase activity in the absence of LDLR. A,B) Plasma lipoprotein lipase contents (A) and lipoprotein lipase activity (B) measured in heparinized C57BL/6J or *Ldlr^−/−^* male mice injected with AAV8‐scramble control or AAV8‐*Srebf2*‐shRNA viruses under chow diet feeding (*n* = 8 mice per group, by two‐way ANOVA). C,D) Plasma lipoprotein lipase contents (C) and lipoprotein lipase activity (D) measured in heparinized *Ldlr^−/−^* male mice injected with AAV8‐scramble control or AAV8‐*Srebf2*‐shRNA viruses under 5‐week chow diet feeding followed by 3‐week western diet feeding (*n* = 6–8 mice per group, by student's *t* test). E) Relative expression of the genes involved in *de novo* lipogenesis, cholesterol synthesis and LPL regulation measured by RNA‐seq using different CRISPR/Cas9‐mediated genetically modified AML12 mouse hepatocyte cell line (*n* = 3 replicates for each cell line). F,G) qPCR expression analysis of genes modulating LPL activity in the liver of C57BL/6J (F) and *Ldlr^−/−^* (G) mice injected with AAV8‐scramble control or AAV8‐*Srebf2*‐shRNA viruses under 5‐week chow followed by 5‐week western diet feeding (*n* = 5–6 mice per group, by student's *t* test). H) qPCR expression analysis of *ANGPTL3*, *ANGPTL4*, and *ANGPTL8* in *SREBF2* knockout and control Huh7 human hepatoma cells (*n* = 3 replicates for each cell line, representative of 3 independent experiments, by student's *t* test). I) qPCR expression analysis of *ANGPTL3*, *ANGPTL4*, and *ANGPTL8* in *SREBF2* knockout or control Huh7 human hepatoma cells lacking LDLR (*n* = 3 replicates for each cell line, representative of 3 independent experiments, by student's *t* test). J–M) Western blot (J and L) and quantification (K and M) of ANGPTL3 (FL stands for full length) and ANGPTL8 in heparinized plasma of 4h‐fasted male C57BL6J mice (J and K, *n* = 7 mice per group, by t student's *t* test) and mice lacking LDLR (L and M, *n* = 6–8 mice per group, by student's *t* test) injected with AAV8‐scramble control or AAV8‐*Srebf2*‐shRNA viruses fed chow. N,O) Western Blot of cellular (N) and secreted (O) ANGPTL3 protein in *SREBF2* knockout and control *LDLR^+/+^* and *LDLR^−/−^* Huh7 human hepatoma cells (representative of 3 independent experiments). P,Q) Western blot and relative quantification of ANGPTL3 in heparinized plasma of 4 h fasted male C57BL/6J mice and mice lacking LDLR injected with AAV8‐scramble control or AAV8‐*Srebf2*‐shRNA viruses fed chow (3 representative mice of each group consisting of 6–8 mice, by one‐way ANOVA). *p < 0.05, **p < 0.01, and ***p < 0.001; Error bars indicate mean ± SD.

Despite hepatic depletion of SREBP2 led to substantially reduced plasma TG/TC levels in both *Ldlr^+/+^* and *Ldlr^−/−^* mice, we observed certain discrepancies in terms of lipid‐lowering efficacy, for instance, plasma TG was reduced by ≈30% in *Ldlr^+/+^* mice, but by ≈60% in *Ldlr^−/−^* mice following SREBP2 depletion (Figure 1). In addition, after western diet feeding for 3 weeks (8 weeks post injection), we only observed significantly reduced VLDL secretion in *Ldlr^+/+^* mice, but not in *Ldlr^−/−^* mice upon SREBP2 depletion, despite the plasma TG/TC‐lowering efficacy in *Ldlr^−/−^* mice is even larger than it in *Ldlr^+/+^* mice. All these taken together suggest that other unknown lipid‐lowering pathways regulated by SREBP2 might be responsible for the discrepancies observed between *Ldlr^+/+^* and *Ldlr^−/−^* mice. Given the fact that plasma TG levels are determined by two key processes‐hepatic production and peripheral lipolysis catabolized by lipoprotein lipase (LPL),^[^ ^19^ ^]^ we next measured plasma LPL levels and activities in heparinized mice. We first found that plasma post‐heparin LPL levels in both *Ldlr^+/+^* and *Ldlr^−/−^* mice fed chow remain unchanged between *Srebf2* knockdown and control mice (**Figure** **3**A). Surprisingly, upon SREBP2 depletion, *Ldlr^+/+^* mice exhibit a 40% decrease in plasma LPL activities compared to control, while *Ldlr* ^−/−^ mice display a 300% increase in plasma LPL activities compared to control, showing completely opposite effects (Figure 3B). After WD feeding for 3 weeks, post‐heparin LPL activities remain significantly higher in *Srebf2* knockdown mice compared to control littermates (Figure 3C,D). These data together suggest that liver SREBP2 plays a distinct regulatory role in regulating peripheral LPL activities, depending on the presence or absence of LDLR. Moreover, plasma TG/TC‐lowering effects in *Ldlr^−/−^* mice upon SREBP2 depletion are likely attributed to both reduced hepatic VLDL secretion rate and promoted peripheral LPL activities. It is also worth noting that post‐heparin peripheral LPL activity in *Ldlr^−/−^* mice is only 30% of the activity observed in *Ldlr^+/+^* mice (Figure 3A,B), indicating that LDLR deficiency has resulted in diminished peripheral LPL activity. This reduction might play a significant role in the development of the hyperlipidemia phenotype observed in LDLR‐deficient mice.

As the rate limiting enzyme for plasma TG clearance and tissue uptake of fatty acids, peripheral LPL activities are largely regulated by liver‐derived extracellular proteins including lipoproteins such as APOA5, APOC2, APOC3, and APOE; as well as angiopoietin‐like proteins such as ANGPTL3, ANGPTL4, and ANGPTL8.^[^ ^7^ ^]^ To explore the possible regulatory mechanisms by which hepatic SREBP2 modulates peripheral LPL activity, we performed transcriptomic profiling of AML12 cells with or without LDLR. In addition to the downregulation of genes related to *de novo* lipogenesis, cholesterol biosynthesis and VLDL assembly in *Srebf2* knockout cells, the mRNA levels of *Angptl3* and *Angptl8* were significantly reduced compared to control cells (Figure 3E). Consistently, mRNA levels of *Angptl3* and *Angptl8* in liver were decreased by 43% and 37%, respectively, following SREBP2 depletion in *Ldlr^+/+^* mice, while they exhibited reductions of 45% and 33%, respectively, in *Ldlr^−/−^* mice upon SREBP2 depletion (Figure 3F,G). Furthermore, we observed that in human hepatocytes, SREBP2 plays similar regulatory roles in the expression of *ANGPTL3* and *ANGPTL8*, irrespective of the presence or absence of LDLR (Figure 3H,I).

We next examined if post‐heparin plasma ANGPTL3 and ANGPTL8 were decreased in the absence of hepatic SREBP2. Surprisingly, despite substantial reduction of mRNA levels of both genes in liver of *Ldlr ^+/+^* mice upon SREBP2 depletion, their protein levels in the blood remain unchanged compared to control littermates (Figure 3J,K). However, in *Ldlr^−/−^* mice, depletion of SREBP2 led to significantly decreased levels of plasma ANGPTL3, while ANGPTL8 levels remain unaffected compared to control littermates (Figure 3L,M). ANGPTL3 is a crucial LPL inhibitor, and it is exclusively expressed in the liver. To examine if the decreased plasma ANGPTL3 levels is due to diminished hepatic secretion in the absence of SREBP2, we measured ANGPTL3 levels in the medium of Huh7 cells. We found that knockout of SREBF2 resulted in a reduction of cellular and secreted ANGPTL3 protein levels exclusively in *LDLR* ^−/−^ Huh7 cells, while such an effect was not observed in *LDLR* ^+/+^ Huh7 cells, despite a similar reduction in mRNA levels in both cell lines (Figure 3N,O). These data suggest that the enhanced peripheral LPL activity observed in *Ldlr^−/−^* mice upon SREBP2 depletion is very likely due to diminished blood ANGPTL3 levels. In addition to its transcriptional regulation of ANGPTL3 expression, SREBP2 appears to mediate an unknown pathway that regulates ANGPTL3 protein homeostasis in hepatocytes with LDLR.

Of note, we found that plasma LPL activity is significantly lower in *Ldlr^−/−^* mice compared to age‐matched C57BL/6J mice (Figure 3B). This reduction is likely attributable to the elevated plasma ANGPTL3 levels observed in *Ldlr^−/−^* mice (Figure 3P,Q). Consistently, LDLR deficiency resulted in a marked increase in secreted ANGPTL3 levels in Huh7 cells (Figure 3N,O).

In addition, we observed that in mice fed with Western diet, which contains higher levels of cholesterol, exhibited significantly increased LPL activity compared to those fed a chow diet (Figure 3B,D). To determine whether this increase in plasma LPL activity was due to elevated plasma ANGPTL3 levels, we compared plasma ANGPTL3 levels between chow‐fed and WD‐fed mice. Both *Ldlr^−/−^* and C57BL/6J mice displayed elevated plasma ANGPTL3 levels under WD feeding compared to chow feeding (Figure S4A–D, Supporting Information). Consistently, cholesterol supplementation in both *LDLR^−/−^* and *LDLR^+/+^* Huh7 cells enhanced ANGPTL3 secretion (Figure S4E–H, Supporting Information). Furthermore, statin treatment slightly reduced ANGPTL3 secretion in *LDLR^−/−^* Huh7 cells (Figure S4I,J, Supporting Information). These results indicated that ANGPTL3 is unlikely to be responsible for the cholesterol‐induced increase in LPL activities. Moreover, the observed elevation in plasma/secreted ANGPTL3 appears to be independent of SREBP2 as high cholesterol feeding increased ANGPTL3 levels in both C57BL/6J mice and *LDLR^+/+^* Huh7 cells, regardless of SREBP2 depletion.

### 2.4 Hepatocyte‐Specific Depletion of SREBP2 Enhances LPL Activity Mainly through ANGPTL3 in the Absence of LDLR

AAV8 is widely used to target liver due to its high efficiency in transducing hepatocytes. However, AAV8‐*Srebf2*‐shRNA also significantly knocks down *Srebf2* in epididymal white adipose tissue (Figure S1C, Supporting Information). To enable hepatocyte‐specific depletion of SREBP2, an AAV8 vector with Cre recombinase driven by Thyroxin Binding Globulin promoter (AAV8‐TBG‐Cre) was used to knock out *Srebf2* in floxed *Srebf2* (*Srebf2^fl/fl^*) mice. Similar to the effects observed in mice with shRNA‐induced *Srebf2* knockdown, hepatocyte‐specific knockout of *Srebf2* also reduced circulating TG and TC levels by 45% and 31%, respectively (Figure S5A,B, Supporting Information), primarily due to decreased VLDL secretion rate (Figure S5C, Supporting Information). Additionally, the loss of hepatocyte SREBP2, in the presence of LDLR, did not affect post‐heparin plasma levels of LPL and ANGPTL3, despite that it significantly reduced peripheral LPL activity (Figure S5D–G, Supporting Information).

**Figure 4:**
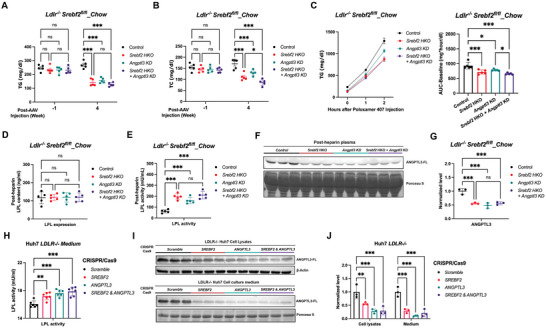
Hepatic SREBP2 regulates peripheral LPL activity mainly through ANGPTL3 in the absence of LDLR. A,B) Plasma triglyceride (A) and cholesterol (B) levels of 16 h fasted *Ldlr^−/−^,Srebf2^fl/fl^* male mice injected with AAV8‐TBG‐GFP or AAV8‐TBG‐Cre together with AAV8‐shRNA‐*scramble* or AAV8‐shRNA‐*Angptl3* to achieve hepatocyte‐specific knockout of *Srebf2* (*Srebf2 HKO*), hepatic depletion of ANGPTL3 (*Angptl3 KD*), and SREBP2 and ANGPTL3 double depletion (*Srebf2 HKO+ Angptl3 KD*) under chow diet feeding (*n* = 5 mice per group, by two‐way ANOVA). C) Changes of plasma triglyceride after Poloxamer‐407 injection in *Ldlr^−/−^,Srebf2^fl/fl^* male mice injected with different AAVs to achieve hepatic depletion of SREBP2 and/or ANGPTL3 under chow diet feeding (*n* = 5 mice per group). VLDL secretion levels were estimated and compared using an area‐under‐the‐curve (AUC) approach that subtracts the baseline value (*n* = 5 mice per group, by two‐way ANOVA). D,E) Plasma total lipoprotein lipase contents (D) and lipoprotein lipase activity (E) measured in heparinized *Ldlr^−/−^,Srebf2^fl/fl^* male mice injected with different AAVs to achieve hepatic depletion of SREBP2 and/or ANGPTL3 under chow diet feeding (*n* = 5 mice per group, by two‐way ANOVA). F,G) Western blot (F) and relative quantification (G) of ANGPTL3 in heparinized plasma of 4 h fasted male *Ldlr^−/−^,Srebf2^fl/fl^* male mice upon SREBP2 and ANGPTL3 single or double depletion under chow diet feeding (3 representative mice of each group consisting of 5 mice, by two‐way ANOVA). H) Activity of LPL pretreated with cell culture medium isolated from CRISPR/Cas9‐mediated SREBF2 and ANGPTL3 single or double knockout or control *LDLR^−/−^* Huh7 human hepatoma cells. (*n* = 3 replicates from individual wells, representative of 3 independent experiments, by two‐way ANOVA). I,J) Western blot (I) and relative quantification (J) of ANGPTL3 in the lysates or culture medium from CRISPR/Cas9‐mediated SREBF2 and ANGPTL3 single or double knockout or control *LDLR^−/−^* Huh7 human hepatoma cells. (*n* = 3 replicates from individual wells, representative of 3 independent experiments, by two‐way ANOVA). *p < 0.05, **p < 0.01, and ***p < 0.001; Error bars indicate mean ± SD.

In the absence of LDLR, the SREBP2‐ANGPTL3‐LPL axis might play a pivotal role in regulating plasma lipid levels. To further investigate if the lipid‐lowering effect upon SREBP2 depletion is due to reduced plasma ANGPTL3 levels in *Ldlr^−/−^* mice, we generated *Srebf2* and *Angptl3* double knockdown mouse models. This was achieved via using AAV‐shRNA to knock down *Angptl3* and using AAV‐TBG‐Cre to deplete SREBP2 in *Ldlr^−/−^, Srebf2^fl/fl^* mice. Under a chow diet feeding, depletion of either SREBP2 or ANGPTL3 individually resulted in ≈47% and ≈42% reductions in plasma TG compared to control littermates. However, double knockdown of *Srebf2* and *Angptl3* did not further reduce plasma TG significantly compared to *Srebf2* knockdown mice (**Figure** **4**A), suggesting that ANGPTL3‐LPL axis is likely the major downstream pathway responsible for reduced plasma TG levels in *Ldlr^−/−^* mice upon SREBP2 depletion. In addition, plasma TC reduction was ≈24% in *Angptl3* knockdown mice but ≈50% in double knockdown mice, indicating a significant contribution of non‐ANGPTL3 effects to cholesterol lowering upon SREBP2 depletion (Figure 4B). Consistently, we next found that VLDL secretion rate was reduced to a larger extent in SREBP2‐depleted mice than it in ANGPTL3‐depleted mice when compared to control mice (Figure 4C).

Acute depletion of hepatic ANGPTL3, a well‐established LPL inhibitor, significantly increased the LPL activity in the heparinized plasma by 260% without affecting LPL content (Figure 4D,E). Furthermore, hepatocyte‐specific depletion of SREBP2 or double depletion of both SREBP2 and ANGPTL3 resulted in a slightly greater increase in post‐heparin LPL activity compared to ANGPTL3 depletion alone (Figure 4E). In line with the increased LPL activity, depletion of SREBP2 and/or ANGPTL3 led to a significant decrease in ANGPTL3 protein levels in heparinized plasma (Figure 4F,G).

To further validate whether ANGPTL3 is the direct downstream mediator of SREBP2 in regulating LPL activity in the absence of LDLR, CRISPR/Cas9 system was used to knock out *SREBF2* and *ANGPTL3* individually or together in human hepatoma Huh7 cells lacking LDLR. Serum‐free cell culture medium was collected to examine its effect on modulating LPL activities in an in vitro assay. Consistent with in vivo findings, both *SREBF2* and *ANGPTL3* knockout resulted in a significant reduction in ANGPTL3 protein levels in the cell lysates and culture medium, which is accompanied with significantly enhanced LPL activity (Figure 4H–J). Moreover, when compared to *ANGPTL3* single knockout, double knockout of *SREBF2* and *ANGPTL3* did not further increase LPL activity, suggesting ANGPTL3 as the main downstream effector of SREBP2 in regulating peripheral LPL activity.

### 2.5 SREBP2 Promotes Proteasome‐Mediated Degradation of ANGPTL3 in the Presence of LDLR

ANGPTL3 has been previously identified as a direct transcriptional target of LXR.^[^ ^11^ ^]^ To investigate if SREBP2 regulates ANGPTL3 levels and LPL activity through LXR, we examined ANGPTL3 levels in *SREBF2* knockout or control *LDLR^+/+^* and *LDLR^−/−^* Huh7 cells treated with T0901317, a validated LXR agonist (Figure S6A, Supporting Information). Consistent with previous studies,^[^ ^11^ ^]^ LXR activation increased ANGPTL3 mRNA levels in Huh7 cells, regardless of LDLR deficiency (Figure S6B,C, Supporting Information). However, SREBP2‐KO cells still exhibited significantly reduced ANGPTL3 mRNA levels in the presence of LXR agonists compared to scramble controls (Figure S6B,C, Supporting Information), suggesting that LXR agonists could not fully restore ANGPTL3 expression in SREBP2‐depleted hepatocytes. In line with transcription levels, LXR activation significantly increased ANGPTL3 secretion in both scramble and SREBP2‐depleted hepatocytes (Figure S6D–G, Supporting Information), but cannot compensate for the loss of SREBP2 in regulating ANGPTL3 secretion and LPL activity in the absence of LDLR (Figure S6F–H, Supporting Information). Notably, conserved sterol regulatory element (SRE) binding sites were identified in the promoter region of ANGPTL3 (Figure S6I, Supporting Information), suggesting that SREBP2 may directly regulate ANGPTL3 transcription in hepatocytes. Collectively, these findings indicate that SREBP2 and LXR regulate ANGPTL3 secretion through at least partially independent mechanisms.

The inconsistent quantitative relations between *ANGPTL3* mRNA and protein levels in the presence or absence of LDLR upon SREBP2 depletion suggest the existence of a SREBP2‐controlled post‐transcriptional regulatory pathway modulating blood ANGPTL3 levels. We next asked how SREBP2 regulates ANGPTL3 protein homeostasis in hepatocytes. Compared to control cells, the elevated ANGPTL3 protein‐to‐mRNA ratio in *LDLR* ^+/+^ hepatocytes upon SREBP2 depletion might be due to increased mRNA stability, elevated translation efficiency, or attenuated protein degradation activities (Figure S6A, Supporting Information).^[^ ^20^ ^]^ We first measured *ANGPTL3* mRNA stability in Huh7 cells by analyzing mRNA half‐life upon inhibition of transcription via Actinomycin D.^[^ ^21^ ^]^ *ANGPTL3* mRNA degradation rate is similar between *SREBF2* knockout and control Huh7 cells with or without LDLR (Figure S7A–C, Supporting Information). As proteasomal proteolysis and lysosomal proteolysis are the most critical pathways responsible for degradation of cellular proteins, we next interrogated if these proteolysis activities are involved in the regulation of ANGPTL3 protein homeostasis. In Huh7 cells, treatment with chloroquine (CQ), a well‐established inhibitor of lysosomal proteolysis, did not significantly affect cellular and secreted ANGPTL3 levels in both wild‐type and *SREBF2* knockout cells in the presence of LDLR (Figure S7D–F, Supporting Information). Moreover, ANGPTL3 levels remain comparable between with and without SREBP2 (Figure S7D, Supporting Information). In *LDLR* ^−/−^ Huh7 cells, CQ treatment resulted in a moderate increase in medium ANGPTL3 levels in both control and SREBF2 knockout cells (Figure S7G–I, Supporting Information). These data suggest that lysosomal proteolysis is not responsible for the elevated ANGPTL3 protein‐to‐mRNA ratio observed in *LDLR* ^+/+^ hepatocytes following SREBP2 depletion.

**Figure 5:**
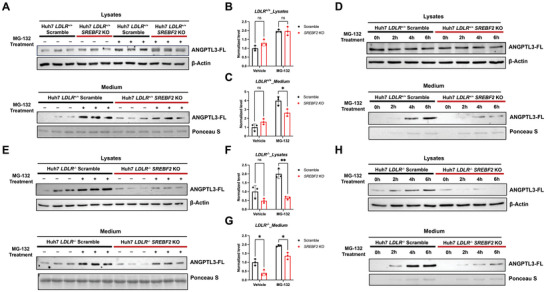
SREBP2 promotes proteasome‐mediated degradation of ANGPTL3 in the presence of LDLR. A–C) Western Blot and quantification of cellular and secreted ANGPTL3 in *SREBF2* knockout or control *LDLR^+/+^* Huh7 cells upon 1 um MG‐132 or DMSO treatment for 4 h. (*n* = 3 replicates from individual wells, representative of 3 independent experiments, by two‐way ANOVA). D) Western Blot of cellular and secreted ANGPTL3 in *SREBF2* knockout or control *LDLR^+/+^* Huh7 cells upon 1 um MG‐132 treatment for 0, 2, 4, and 6 h (representative of 3 independent experiments). E–G) Western Blot and quantification of cellular and secreted ANGPTL3 in *SREBF2* knockout or control *LDLR^−/−^* Huh7 cells upon 1 um MG‐132 or DMSO treatment for 4 h. (*n* = 3 replicates from individual wells, representative of 3 independent experiments, by two‐way ANOVA). H) Western Blot of cellular and secreted ANGPTL3 in *SREBF2* knockout or control *LDLR^−/−^* Huh7 cells upon 1uM MG‐132 treatment for 0, 2, 4, and 6 h (representative of 3 independent experiments). *p < 0.05, **p < 0.01, and ***p < 0.001; Error bars indicate mean ± SD.

Next, we subjected Huh7 cells to treatment with the proteasomal proteolysis inhibitor MG‐132. Our data in *LDLR* ^+/+^ hepatocytes indicate that, when compared to vehicle, scramble control cells treated with MG‐132 exhibited a significantly greater increase in secreted ANGPTL3 levels compared to SREBP2‐deficient cells (**Figure** **5**A–C). A time course treatment of scramble cells with MG‐132 also showed a more rapid increase in medium ANGPTL3 levels compared to SREBP2 depleted cells (Figure 5D). These data suggest that SREBP2 in *LDLR* ^+/+^ hepatocytes promotes both ANGPTL3 transcription and proteasomal proteolysis‐mediated protein degradation. While depletion of SREBP2 in *LDLR* ^+/+^ hepatocytes significantly reduces the mRNA levels of ANGPTL3, the diminished proteasome activity toward ANGPTL3 keeps its protein levels unaffected.

In hepatocytes lacking LDLR, this SREBP2‐proteasome‐ANGPTL3 pathway seems to be abolished or greatly attenuated as evidenced by the observation that MG‐132 treatment led to a similar degree of increase of both cellular and secreted ANGPTL3 levels between *SREBF2* knockout and control cells (Figure 5E–H).

### 2.6 Acute Depletion of Hepatic SREBP2 Shows a Synergistic Cholesterol Lowering Effect with PCSK9 Inhibition

**Figure 6:**
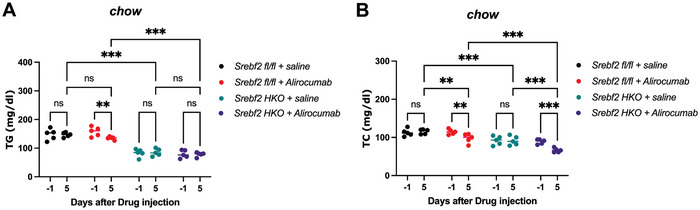
Acute depletion of hepatic SREBP2 synergizes with PCSK9 inhibition to reduce plasma cholesterol levels. A,B) Plasma triglyceride (A) and cholesterol (B) levels of *Srebf2^fl/fl^* and *Srebf2* hepatocyte‐specific knockout (*Srebf2 HKO*) mice administrated with single‐dose Alirocumab or saline (*n* = 5 mice per group, by two‐way ANOVA). *p < 0.05, **p < 0.01, and ***p < 0.001; Error bars indicate mean ± SD.

PCSK9 inhibitors, including monoclonal antibodies and siRNA therapies, prevent the degradation of LDLR on hepatocyte surfaces, showing remarkable efficacy in reducing LDL‐C and atherosclerotic events in patients with familial hypercholesterolemia.^[^ ^22^ ^]^ Previous studies have shown that hepatocyte‐specific deletion of *Srebf2* did not affect liver LDLR protein levels,^[^ ^15^ ^]^ suggesting that PCSK9 inhibition might have additive effects on cholesterol reduction when combined with hepatic SREBP2 depletion. To determine the potential synergistic effects, Alirocumab, an FDA‐approved PCSK9 monoclonal antibody, was injected to mice with hepatocyte‐specific SREBP2 depletion. Consistent with previous findings, hepatocyte‐specific knockout of *SREBF2* resulted in the reduction of plasma TG (**Figure** **6**A) and TC (Figure 6B) by 47% and 21%, respectively, compared to the control littermates. A single‐dose injection of Alirocumab did not further reduce circulating TG in mice lacking hepatic SREBP2 (Figure 6A), but significantly enhance the cholesterol‐lowering effects from 21% to 43% (Figure 6B), compared to the control littermates without SREBP2 or PCSK9 inhibition. These data indicate that acute depletion of hepatic SREBP2 works synergistically with PCSK9 inhibitors to lower plasma cholesterol levels.

### 2.7 Acute Depletion of Hepatic SREBP2 Protects Against Atherosclerosis

**Figure 7:**
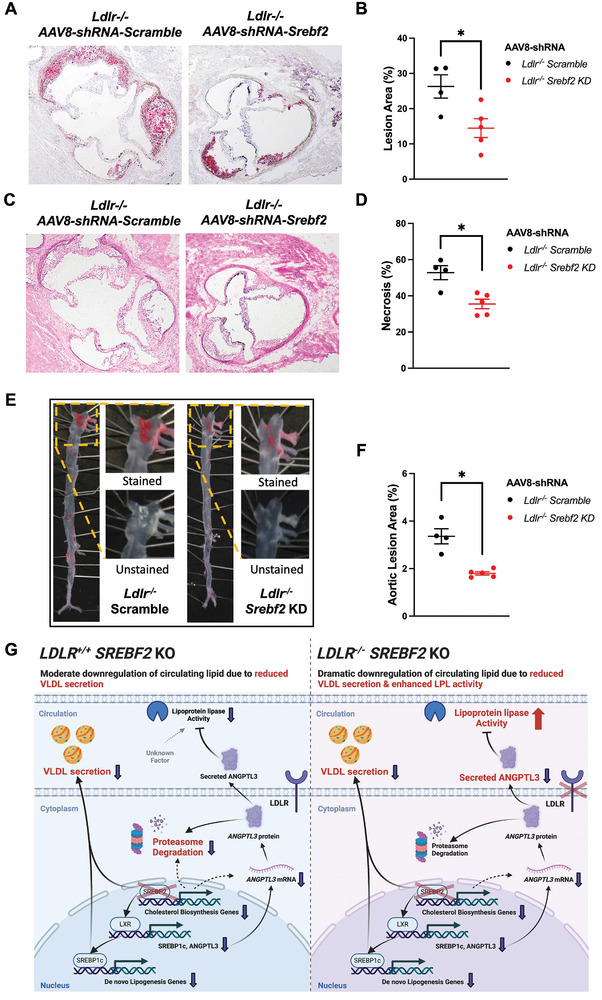
Acute depletion of hepatic SREBP2 protects against atherosclerosis. A,B) Representative images (A) and quantification of aortic lesion areas (B) from cross sectional aortic root sections stained with Oil Red O and hematoxylin for lesion area from LDLR‐Deficient mice injected with AAV8‐scramble control or AAV8‐*Srebf2*‐shRNA and fed western diet for 14 weeks (*n* = 4–5 mice per group, each mouse has at least 3 sections for analysis, by student's *t* test). C,D) Representative images (C) and quantification of necrosis level (D) from cross sectional aortic root sections stained with hematoxylin and eosin for lesion area from LDLR‐Deficient mice injected with AAV8‐scramble control or AAV8‐*Srebf2*‐shRNA and fed western diet for 14 weeks (*n* = 4–5 mice per group, each mouse has at least 3 sections for analysis, by student's *t* test). E,F) Representative images (E) and quantification of aortic lesion areas (F) from *en face* analysis of aortas before and after Oil Red O staining in the male *Ldlr^−/−^* mice injected with AAV8‐scramble control or AAV8‐*Srebf2*‐shRNA and fed with western diet for 14 weeks (*n* = 4–5 mice per group, by student's *t* test). G) Model of the mechanism by which hepatic SREBP2 differentially regulates ANGPTL3 and peripheral LPL activity in the presence or absence of LDLR. *p < 0.05, **p < 0.01, and ***p < 0.001; Error bars indicate mean ± SD.

To determine whether the lipid‐lowering effect upon SREBP2 acute depletion protects mice against atherosclerosis, we examined atherosclerotic plaques in mice lacking LDLR. Analyses of aortic root sections showed that compared to *Ldlr^−/−^* mice injected with AAV8‐shRNA scramble control, the mean lesion area and necrotic core area were reduced by ≈45% and ≈33%, respectively, after one‐dose AAV‐shRNA based depletion of SREBP2 (**Figure** **7**A–D; Figure S8B,C, Supporting Information). Meanwhile, *en face* Oil Red O staining of the full‐length aorta indicated that the atherosclerotic lesion area was also significantly reduced by ≈47% (Figure 7E,F; Figure S8A, Supporting Information). Taken together, these data suggest that acute depletion of SREBP2 in liver of adult mice confers protection against hyperlipidemia and atherosclerosis.

## 3 Discussion

Our results show that acute depletion of SREBP2 specifically in hepatocytes reduces plasma cholesterol and TG levels substantially and confers significant protection to LDLR‐deficient mice against atherosclerosis. Mechanistically, we uncovered that SREBP2 plays a distinct regulatory role in regulating blood ANGPTL3 levels and peripheral LPL activities, depending on the presence or absence of LDLR. Depletion of SREBP2 drastically elevates LPL activities through downregulation of plasma ANGPTL3 levels in LDLR‐deficient mice, but not in C57BL/6J wild‐type mice. This study thus uncovered a liver‐peripheral crosstalk mediated by SREBP2‐ANGPTL3 axis and suggests that SREBP2 inhibition could be an effective strategy to tackle homozygous familial hypercholesterolemia (HoFH).

Homozygous familial hypercholesterolemia (HoFH) is a rare life‐threatening genetic disorder characterized by substantially elevated levels of low‐density lipoprotein cholesterol (LDL‐C) in the bloodstream, which usually is accompanied by premature atherosclerotic cardiovascular disease (ASCVD).^[^ ^5^ ^]^ Genetic studies have revealed that more than 95% of individuals with HoFH carry loss‐of‐function mutations in both alleles of *LDLR* gene.^[^ ^23^ ^]^ Previous studies have established that hypercholesterolemia found in HoFH patients is attributed to greatly attenuated hepatic clearance of plasma LDL‐C, as well as overproduction of cholesterol in the liver due to the loss of feedback inhibition of SREBP2‐mediated cholesterol biosynthesis. In this study, we observed that peripheral LPL activity is significantly reduced in LDLR‐deficient mice compared to wild‐type C57BL/6J mice, which likely contributes to the hyperlipidemia phenotype. It is possible that the upregulation of hepatic SREBP2 activity in LDLR‐deficient hepatocytes increases the expression of ANGPTL3 (Figure 3N–Q), thereby inhibiting peripheral LPL activity.

Previous studies have demonstrated the essential role of LDLR in the liver to communicate with blood thereby regulating hepatic cholesterol production via SREBP2‐mediated signaling pathway.^[^ ^12^ ^]^ In addition to liver, the enzyme lipoprotein lipase in peripheral tissues also plays a major role in regulating cholesterol and lipid homeostasis. Given the essential roles of hepatic SREBP2‐LDLR axis and peripheral LPL in regulating systemic cholesterol and lipid metabolism, it is possible that a mechanism exists for facilitating crosstalk between these two pathways, thus ensuring the maintenance of whole‐body cholesterol/TG homeostasis. In LDLR‐deficient mice, we have identified hepatic ANGPTL3 as the mediator enabling SREBP2 to communicate with peripheral LPL‐mediated hydrolysis of TG‐rich lipoproteins. The study of SREBP2 and ANGPTL3 double depletion in LDLR‐deficient mice revealed that while the TG‐lowering effects upon SREBP2 depletion are primarily driven by the downregulation of ANGPTL3, there may be additional non‐ANGPTL3‐related contributions to the reduction in plasma cholesterol in SREBP2‐deficient mice. Notably, the hepatic VLDL secretion rate is reduced to a larger extent in SREBP2‐deficient mice than in ANGPTL3‐deficient mice when compared to control mice (Figure 4C). These findings suggest that SREBP2 regulates plasma TC levels through both the ANGPTL3‐LPL axis and hepatic VLDL production, as well as potentially through other unknown pathways. Importantly, our study also confirmed that the depletion of hepatic ANGPTL3 inhibits VLDL secretion, suggesting that ANGPTL3 regulates circulating TG and TC levels not only by modulating peripheral LPL activity but also by regulating cellular lipoprotein metabolism.^[^ ^24^, ^25^ ^]^

This SREBP2‐ANGPTL3‐LPL axis functions under disease condition where LDLR is lacking. However, in *wild‐type* mice under physiological conditions, the detailed molecular mechanism underlying reduced peripheral LPL activity following SREBP2 depletion remains unclear. This regulatory effect on LPL activity by SREBP2 is a plausible way for the liver to reduce the uptake of fatty acids by peripheral tissues when lipid production is decreased. It seems that ANGPTL3 is not involved, as we did not observe a significant increase in its protein levels in the blood. Nevertheless, we did notice a significant downregulation in the expression of *Apoa5*, which encodes a critical LPL activator, in both *Ldlr^+/+^* and *Ldlr^−/−^* mice following SREBP2 depletion (Figure 3F,G). Further studies are necessary to investigate whether SREBP2 regulates peripheral LPL activity through APOA5.

It appears that SREBP2 regulates hepatic ANGPTL3 levels through both transcriptional and post‐translational mechanisms. In hepatocytes with LDLR, depletion of SREBP2 results in the downregulates of both ANGPTL3 transcription and proteasome‐mediated protein degradation. Consequently, this leads to no changes in the total protein levels of ANGPTL3. We found that LDLR is not involved in SREBP2's transcriptional regulation of ANGPTL3. However, the inhibitory effect on ANGPTL3 degradation upon SREBP2 knockout is abolished or significantly attenuated by the depletion of LDLR, which restores the correlation between ANGPTL3 mRNA and protein levels.

Our findings strongly support a direct role for SREBP2 in regulating proteasome‐mediated ANGPTL3 degradation in hepatocytes expressing LDLR; however, the underlying molecular mechanism remains unclear. One potential explanation is the existence of a specific E3 ligase, transcriptionally regulated by SREBP2, that recognizes ANGPTL3 as its substrate in hepatocytes. Alternatively, as a master transcriptional regulator of lipid metabolism, SREBP2 depletion alters the expression of many genes and downstream biological processes. Consequently, ANGPTL3 degradation might be secondary to pathways downstream of SREBP2. Although our data suggest that reduced ANGPTL3 degradation upon SREBP2 depletion is not attributable to changes in cellular cholesterol levels (Figure S4C,D,G,H, Supporting Information), the possibility that ANGPTL3 degradation is mediated or modulated by other secondary pathways downstream of SREBP2 cannot be excluded. Future studies are required to elucidate this complex regulatory network, including the identification of specific E3 ligases and other indirect regulators downstream of SREBP2, to fully understand the mechanisms underlying the proteasome‐mediated degradation of ANGPTL3.

Of note, regulation of LDL receptor by SREBP2 is the dominant mechanism by which statin drugs lower plasma LDL‐cholesterol levels. Statins, upon untaken by the liver, block cholesterol production by inhibiting the enzymatic activity of HMG‐CoA reductase. Subsequently, the depletion of cellular cholesterol levels activates SREBP2, leading to an increase in LDL‐cholesterol uptake through the upregulation of LDLR expression in the liver cells. However, patients with HoFH exhibit a significantly diminished response to statins due to the defect or complete loss of LDLR activity. Thus, development of LDLR‐independent cholesterol‐lowering therapies is still urgently needed to address this unmet medical need for the treatment of HoFH. Of note, while our study demonstrated that acute depletion of SREBP2 in C57BL/6J wild‐type and LDLR‐deficient mice did not result in excessive lipid accumulation, increased neutrophil infiltration, or elevated pro‐inflammatory cytokine expression, concerns remain regarding the development of therapies targeting SREBP2. Given the central role of SREBP2 in cholesterol and lipid metabolism, the long‐term side effects of liver SREBP2 inhibition, if there is any, remain unknown. Addressing these concerns will be critical for the successful development of SREBP2‐targeted therapies for HoFH.

In summary, our findings demonstrate that acute depletion of hepatic SREBP2 protects against hypercholesterolemia and atherosclerosis. We elucidated distinct functions of SREBP2 in regulating ANGPTL3 expression and peripheral LPL activities in the presence and absence of LDLR. This study has also provided insights into a long‐standing question: whether the lipid‐lowering effects upon SREBP2 depletion requires intact LDLR. These data taken together suggest that SREBP2 inhibition could serve as an effective strategy to address the challenges posed by HoFH.

## 4 Experimental Model and Subject Details

### 4.1 *E. coli* Strain

To reduce the frequency of homologous recombination of long terminal repeat (LTRs), *E. coli* stbl3 strain cultured in LB broth was used to clone all of the lentiviral vectors.

### 4.2 Animal Models

All animal care and experimental procedures utilized in this study were granted approval by the Institutional Animal Care and Use Committee of the National University of Singapore. Mice were housed in a controlled environment with a 12 h light‐dark cycle, ensuring free access to water and a standard chow diet. *C57BL/6J* (The Jackson Laboratory, RRID: IMSR_JAX:000664) and *Ldlr^−/−^* mice (The Jackson Laboratory, RRID: IMSR_JAX:01 0803) were procured from Jackson Laboratory at 8 weeks of age. Floxed *Srebf2* (*Srebf2^fl/fl^*) mice were gifts from Dr. Ling Shuo‐Chien, Department of Physiology, NUS Yong Loo Lin School of Medicine. *Srebf2^fl/fl^* mice were crossed with *Ldlr^−/−^* mice to generate *Ldlr^−/−^*/*Srebf2^fl/fl^* mice. Male littermate mice aged between 6 to 22 weeks were selected to ensure matching age and sex. In experiments involving special diets, 7‐week‐old mice were placed on a high‐fat diet (60/Fat, TD.06414, Envigo) for a total duration of 10 weeks or a western diet (TD.10885, Envigo) for a total period of 10–14 weeks.

### 4.3 Cell Lines and Culture Conditions

The Huh7 cell line is a hepatocyte‐derived carcinoma cell line that originated from a liver tumor in a 57‐year‐old Japanese male (JCRB Cell Bank, JCRB0403). The AML12 (alpha mouse liver 12) cell line was established from hepatocytes obtained from a male mouse (CD1 strain, line MT42) transgenic for human TGF alpha (ATCC, ATCC CRL‐2254). HEK293T cells were derived from human embryonic kidney 293 cells expressing SV40 T‐antigen (ATCC, ATCC CRL‐3216). Huh7 and HEK293T cells were cultured in Dulbecco's Modified Eagle's Medium (DMEM) supplemented with 10% fetal bovine serum (FBS) and 1% penicillin/streptomycin. AML12 cells were cultured in Dulbecco's Modified Eagle's Medium/Nutrient Mixture F‐12 Ham with 10% FBS, 1% penicillin/streptomycin, 0.005 mg mL^−1^ insulin, 0.005 mg mL^−1^ transferrin, 5 ng mL^−1^ selenium, and 40 ng mL^−1^ dexamethasone. All cells were maintained in a 5% CO_2_ atmosphere at 37 °C.

## 5 Experimental Section

### In Vivo Depletion of Hepatic *Srebf2* and *Angptl3* Using AAV‐Delivered shRNA or AAV‐TBG‐Cre

To knock down hepatic *Srebf2* in vivo, AAV8‐*Srebf2*‐shRNA (Vector Biolabs) were administered to six‐week‐old C57BL/6J, *Ldlr^−/−^* mice via retro‐orbital injection at a dose of 6 × 10^11^ gc per male mouse, same dose of AAV8‐scramble‐shRNA (Vector Biolabs) was used as the control. To achieve hepatocyte‐specific knockout of *Srebf2* in vivo, AAV8‐TBG‐Cre were administered to six‐week‐old *Srebf2^fl/fl^ or Ldlr^−/−^*/*Srebf2^fl/fl^* mice via retro‐orbital injection at a dose of 3 × 10^11^ gc per male mouse, same dose of AAV8‐TBG‐EGFP was used as the control.

To perform *Srebf2* and *Angptl3* double knockdown experiments, age matched *Ldlr^−/−^*/*Srebf2^fl/fl^* mice were randomly divided into 4 groups: Group 1 (control): received 3 × 10^11^ gc AAV8‐TBG‐EGFP and 3 × 10^11^ gc AAV8‐scramble‐shRNA; Group 2 (hepatocyte‐specific *Srebf2* knockout, *Srebf2 HKO)*: received 3 × 10^11^ gc AAV8‐TBG‐Cre and 3 × 10^11^ gc AAV8‐scramble‐shRNA; Group 3 (*Angptl3 knockdown, Angptl3 KD)*: received 3 × 10^11^ gc AAV8‐TBG‐EGFP and 3 × 10^11^ gc AAV8‐*Angptl3*‐shRNA; Group 4 (hepatocyte‐specific *Srebf2* knockout + *Angptl3 knockdown, Srebf2 HKO + Angptl3 KD)*: received 3 × 10^11^ gc AAV8‐TBG‐Cre and 3 × 10^11^ gc AAV8‐*Angptl3*‐shRNA. AAV8 administration was performed via retro‐orbital injection at the age of 6–8 weeks, special diet‐feeding was initiated at either 1‐ or 6‐weeks post‐injection. Heparinized blood and liver samples were collected at specified time points after injection for further analysis.

### Blood and Cellular Lipid Assays

Mouse blood was collected from tail tips at different conditions as specified, and plasma were further isolated via 10 min 1500 × g centrifugation at 4 °C. Cellular lipids of mouse liver or cultured cell lines were extracted using Folch's extraction method (Folch et al., 1957) with optimization. In brief, snap‐frozen liver samples or cell pellets suspended in PBS were mixed with six‐fold volume 2:1 (v:v) chloroform‐methanol mixture and vortexed vigorously for at least 30 s. Then 75 ul of PBS was added, followed by another round of vigorous vortex. The mixture was centrifuged at 4200 × g for 10 min at 4 °C, 200 ul of the organic layer (bottom phase) was transferred into a clean tube to dry overnight. The dried residue was dissolved in 100 ul ethanol containing 1% Triton X‐100 and dried using a SpeedVac concentrator. The final dried cellular lipids were dissolved in 100 ul 1% Triton X‐100 in PBS and proceeded for analysis. Triglyceride and total cholesterol levels were measured using Infinity Triglycerides Reagent (Thermo Fisher) and Infinity Cholesterol Reagent (Thermo Fisher) respectively according to the manufacturer's instructions.

### CRISPR‐Induced Depletion of SREBP2, LDLR, and ANGPTL3 in Human Hepatoma Huh7 Cells and Mouse AML12 Cells

Gene knockout experiments using CRISPR/Cas9 were performed in human Huh7 cells and mouse AML12 cells, following a modified protocol from a previous study (Yu et al., 2019). In brief, stable cell lines expressing Cas9 were generated in Huh7/AML12 cells through a two‐round infection of packaged in lentiviruses containing lentiCas9‐Blast (Addgene) for 16 h, followed by selection with 30 µg ml^−1^ blasticidin (ThermoFisher, A1113903) for 5 days. Subsequently, three guide RNAs targeting either human *SREBF2* or mouse *Srebf2* were constructed into lentiGuide‐Puro (Addgene). The lentiviruses containing corresponding CRISPR lentiviral vectors were packaged from HEK293FT cells and used to infect Huh7 2xCas9 cells or AML12 2xCas9 cells for 24 h, followed by selection using 10 µg ml^−1^ puromycin (ThermoFisher, A1113803) for five days prior to conducting assays.

### In Vitro APOB100 Secretion Assay

Huh7 cells with different CRISPR‐mediated gene depletion were seeded 24 h prior to the assay for recovery, followed by a 12 h culture in serum‐free DMEM medium. Subsequently, the medium was collected and the quantity of secreted APOB100 was measured using ELISA kit (ThermoFisher, EH34RB) in accordance with the manufacturer's instructions.

### In Vitro LDL Uptake Assay

To measure LDL uptake, Huh7 cells were seeded 16 h prior to the assay. Then, the cells were rinsed once with PBS and incubated in serum‐free DMEM medium for 4 h before 1 h incubation in serum‐free medium containing 5 µg mL^−1^ of Dil‐LDL (Thermo Fisher). The cells were then carefully washed with PBS to remove unbound or surface‐bound LDL and fixed with 4% paraformaldehyde and co‐stained with DAPI for imaging. In parallel, cells were collected to perform flow cytometry analysis to quantify the mean fluorescence intensity (MFI) as a measure of LDL uptake by the cells.

### Hepatic VLDL Secretion Assay

To measure the hepatic VLDL secretion rate, mice were fasted for 4 h. Subsequently, they received an intraperitoneal injection of 1g kg^−1^ body weight Poloxamer‐407 (Sigma). Immediately before, at 60 min, and 120 min after the administration of Poloxamer‐407, blood samples were collected from the tail tips. Plasma triglyceride levels were measured using the Infinity Triglycerides Reagent (Thermo Fisher) as mentioned previously. VLDL secretion rate was estimated and compared using an area‐under‐the‐curve (AUC) approach that subtracts the baseline value.

### Lipoprotein Lipase Activity and ELISA Assay

The activity of lipoprotein lipase (LPL) was assessed in the fasting post‐heparin plasma using a fluorometric kit (Cell Biolabs, STA‐610). Prior to and 10 min after the retro‐orbital injection of 0.1U heparin per gram of the mouse's body weight, blood was collected from the tail vein into heparinized tubes. The plasma was then separated and processed for the assay in accordance with the manufacturer's instructions. Fluorescence was measured using a microplate reader at an excitation wavelength of 485 nm and an emission wavelength of 525 nm. LPL activity was calculated and reported as mU ml^−1^. The circulating lipoprotein lipase protein levels in the mouse post‐heparin plasma were determined by a mouse‐specific ELISA kit (Cusabio, CSB‐E08495 m) in accordance with the manufacturer's instructions.

### Immunoblotting

Tissue or cell samples were lysed in RIPA lysis buffer consisting of 50 mm Tris‐HCl (pH 7.5), 150 mm NaCl, 1 mm EDTA, 1 mm DTT, 1% (v/v) Nonidet P‐40, 0.5% (w/v) sodium deoxycholate, 0.1% (w/v) SDS, and a protease and phosphatase inhibitor cocktail (Thermo Fisher, 78443). Protein concentrations were determined using the BCA Protein Quantification Kit (Thermo Fisher, 23227). Proteins (20–50 µg) were then subjected to 8–12% SDS‐PAGE and transferred to a Nitrocellulose Membrane (Bio‐rad, 1 620 094) for immunoblot analysis. After blocking the membrane with 5% (w/v) milk in TBST (Tris‐buffered saline with 0.1% Tween 20), it was incubated overnight with the primary antibody, followed by three washes with TBST. Subsequently, the membrane was incubated with the corresponding secondary HRP‐conjugated antibody diluted in TBST containing 5% milk (w/v). After three additional washes with TBST, the bands on the membrane were detected using the iBright FL1500 system (Thermo Fisher, FL1500).

For immunoblotting with mouse plasma, equal amounts of the plasma were diluted with PBS for 5–20 folds and mixed with loading buffer before proceeding to the SDS‐PAGE. Membrane was stained with Ponceau S and the total protein stain was used as the loading control for quantification.

### Gene Expression Analysis

For mRNA expression analysis, total RNA was isolated using Trizol (Invitrogen, 15596018) and the expression levels of specific genes were measured by quantitative real‐time PCR (qRT‐PCR) using High‐Capacity cDNA Reverse Transcription Kit (Thermo Fisher, 4 368 813) and SYBR‐green qPCR master‐mix (Qiagen, 208057) with the recommended setup. For the reverse transcription, the thermocycler conditions were 25 °C 10 min, 37 °C 120 min, and 85 °C 5 min. For the quantitative PCR, the thermocycle was 95 °C for 5 min, followed by 40 cycles of 95 °C (denaturation) for 10 s and 60 °C (annealing and extension) for 30 s. The melting curve was determined by one‐cycle 95 °C for 15 s and 60 °C for 1 min at last. The expression level was quantified using the ΔCt method, standardized against the Ct value of a housekeeping gene RPLP0. Primer sequences used in this study were listed in Table S1 (Supporting Information).

### RNA‐seq and Data Analysis

For the RNA‐seq assay, total mRNA was isolated from AML12 cell samples using Trizol (Invitrogen, 15 596 018). The extracted total RNA was quantified using a Nanodrop 2000 spectrophotometer (Thermo Fisher) and assessed for quality using a Bioanalyzer. cDNA library preparation and mRNA sequencing were performed by Novogene, utilizing the state‐of‐the‐art Illumina NovaSeq platforms and a paired‐end 150 bp sequencing strategy.

For data analysis, the raw reads were initially processed with FastQC and Cutadapt to eliminate adapters, over‐represented sequences, and low‐quality sequences. Subsequently, the alignment of the reads was conducted using the HISAT2 software. SAMtools was utilized for sorting and converting the mapped reads to the BAM format. Following that, reads per kilobase per million (RPKM) values were calculated for each gene, and differentially expressed genes (DEGs) were identified using DESeq2, considering those with a fold change of more than 1.5 and an adjusted P value of less than 0.05. To further explore the biological implications of the DEGs, Gene Ontology (GO) biological process term enrichment analysis was carried out employing the clusterProfiler package. Gene sets with a P value below 0.05 were considered statistically significant.

### In Vitro Lipoprotein Lipase Activity Assay

To evaluate the regulatory activity of secretory factor from the hepatocytes, an in vitro LPL activity assay was used as described^[^ ^26^ ^]^ with modifications. Briefly, 25 ul serum‐free overnight cell culture medium from Huh7 cells was diluted to 100 ul using 1xLPL Assay buffer containing 15 mU ml^−1^ LPL from the commercial assay kit (Cell Biolabs, STA‐610). The reaction was first carried out for 30 min at 37 °C and proceeded in accordance with the manufacturer's instructions. The signal of the fluorescent product was measured using a microplate reader at an excitation wavelength of 485 nm and an emission wavelength of 525 nm.

### RNA Stability Analysis

Huh7 cells were incubated with 1 ug ml^−1^ Actinomycin D (dissolved in DMSO) for different time courses (0, 3, 6, 12, and 24 h) to inhibit RNA synthesis. Cultures that were treated with DMSO only served as the vehicle control. Subsequently, gene expression was determined and normalized to GAPDH, a housekeeping gene with stable transcripts. *Myc*, whose mRNA has an extreme instability in various cell types (Dani et al., 1984), was used as a positive control for the transcription inhibition.

### Protein Degradation Inhibitor Treatment

Cells (5 × 10^5^) were seeded in 6‐well plates 24 h prior to the treatment to let them fully adhere to the surface and present healthy phenotype. After recovery, cells were first starved in serum‐free DMEM medium for 4 h and then changed into the serum‐free medium supplementary with different inhibitors (1 um Proteasome inhibitor MG‐132 dissolved in DMSO (MedChemExpress, HY‐13259) or 50 um Lysosomal autophagy inhibitor Chloroquine dissolved in ethanol (MedChemExpress, HY‐17589A)) or equal volume of the solvents as the vehicle control for treatment of various lengths (0, 2, 4, and 6 h). When the treatment terminated, both cell lysates and culture medium were collected for immunoblotting.

### LXR Agonist, Cholesterol, and Statin Treatment

Cells (5 × 10^5^) were seeded in 6‐well plates 24 h prior to the treatment to let them fully adhere to the surface and present healthy phenotype. After recovery, cells were first starved in serum‐free DMEM medium for 4 h and then changed into the serum‐free medium supplementary with different chemicals or supplements (1uM LXR agonist T0901317 dissolved in DMSO (MedChemExpress, HY‐10626) or 50 um Cholesterol–methyl‐β‐cyclodextrin dissolved in H2O (Sigma‐Aldrich, C4951) or 10 um Simvastatin (MedChemExpress, HY‐17502)) or equal volume of the solvents as the vehicle control for overnight treatment (16 h). When the treatment terminated, both cell lysates and culture medium were collected for immunoblotting.

### Synergistic Effect of PCSK9 Inhibitors and SREBP2 Depletion In Vivo

To evaluate the synergistic effect of PCSK9 inhibition and SREBP2 depletion. Six‐week‐old *Srebf2^fl/fl^* mice injected with AAV8‐TBG‐EGFP or AAV‐TBG‐Cre were maintained on chow diet. Alirocumab (10 mg kg^−1^) was administered via tail vein injections after three weeks of AAV injection. Immediately before and 120 h after Alirocumab injection, overnight fasted blood samples was collected from tail tips for lipid analysis.

### En Face Quantification of Atherosclerotic Lesions in Aorta

Seven‐week‐old *Ldlr* ^−/−^ mice injected with AAV8‐scramble‐shRNA (Vector Biolabs) or AAV8‐Gpr146‐shRNA (Vector Biolabs) were placed on western diet (TD.10885, Envigo) for a total period of 14 weeks. At the end of the dietary intervention, the mice were anesthetized, and their aorta was carefully perfused with 10 mL of saline via the left ventricle. The heart, along with the attached full‐length aorta, was then carefully detached and fixed in 10% formalin for a duration of 3 days. Following removal of the heart and surrounding adventitial fat tissue under a dissection microscope, the aorta was longitudinally opened from the aortic root to iliac bifurcation and pinned on a black rubber plate. To visualize the plaques, the aorta was treated with 60% isopropanol for 10 min before staining with an oil red O solution (3 mg mL^−1^ in 60% isopropanol, filtered twice through a 0.2 µm filter) for 15 min. After staining, the aorta was destained with 60% isopropanol for 5 min to eliminate background staining. The stained aortas were then stored in 10% formalin until images were captured. Quantification of the oil‐red O‐stained atherosclerotic lesion area in the full‐length aorta was performed using ImageJ software. All image capture and quantitation procedures were conducted in a blinded manner.

### Histology and Immunohistochemistry

Mouse hearts were perfused in situ with 0.9% w/v saline via a cannula inserted into the left ventricle, with outflow through an incision in the right atrium. The perfused hearts were then embedded fresh in cryo‐embedding media (Tissue‐Tek OCT compound). Frozen tissue blocks were mounted on a cryostat mold, ensuring that the tip of the heart faced outward, and sectioning commenced until the appearance of the first aortic valve. Subsequently, serial sections were collected at a thickness of 10 micrometers per section, covering the complete sequence of 2 aortic valves, followed by 3 aortic valves, and finally 1 aortic valve. For individual mouse, one aortic root sections containing at least 2 aortic valves were stained with Oil Red O and counterstained with hematoxylin for lesion area analysis. A consecutive section was stained with Hematoxylin and Eosin for necrosis quantification. Quantification of both the lesion area and necrosis level was performed using ImageJ software. The lesion area fraction was calculated by dividing the lesion area (Oil Red O positive area) by the area of the aortic wall and expressed as a percentage. The necrosis level was determined by dividing necrotic core areas (defined as anuclear, afibrotic, and eosin‐negative areas) by the lesion area and expressed as a percentage.

Mouse livers were fixed in 4% paraformaldehyde for 48 h followed by gradual dehydration in a series of alcohol solutions with increasing concentrations. Subsequently, the liver samples were cleared in xylene, infiltrated with liquid paraffin, and embedded in paraffin blocks for sectioning. Sections with 4 micrometers thick were cut and mounted onto glass slides. After deparaffinization and rehydration, the slides were stained with hematoxylin and counterstained with eosin for further observation and analysis. The steatosis level was graded using a standardized NAFLD Activity Score (NAS). All quantification and scoring were conducted in a blinded manner.

### Quantification and Statistical Analysis

All statistical analyses were performed using GraphPad Prism 9.3. Data were expressed as the mean ± standard deviation using at least three biologically independent replicates. The statistical differences between the two experimental groups were calculated using a heteroscedastic two‐tailed student's *t*‐test. For comparisons among multiple groups, one‐way analysis of variance (ANOVA) was used, followed by Tukey's or Dunnett's post hoc test, as appropriate. For experiments involving two independent variables, two‐way ANOVA was employed to evaluate interactions and main effects, with Bonferroni's post hoc tests applied for pairwise comparisons where necessary. A p‐value of <0.05 was considered statistically significant.

## Conflict of Interest

The authors declare no conflict of interest.

## Author Contributions

H.Y. performed conceptualization, supervision, and funding acquisition. Y.W., Y.S., and H.Y. performed methodology. Y.W., J.C., and H.Y. performed formal analysis. Y.W., Y.S., J.C., T.T., X.C., K.C., H.L., Y.J., Y.L., and H.Y. performed investigation. Y.W. and H.Y. performed wrote the original draft. M.A., R.F., Y.S., and H.Y. provided resources.

## Supporting information



Supporting Information

Supplemental Table 1

## Data Availability

The data that support the findings of this study are available from the corresponding author upon reasonable request.

## References

[1] R. A. Hegele , Nat. Rev. Genet. 2009, 10, 109. 19139765 10.1038/nrg2481

[2] J. D. Horton , J. L. Goldstein , M. S. Brown , J. Clin. Invest. 2002, 109, 1125. 11994399 10.1172/JCI15593 PMC150968

[3] S. Kersten , Biochim. Biophys. Acta 2014, 1841, 919. 24721265 10.1016/j.bbalip.2014.03.013

[4] J. R. Mead , S. A. Irvine , D. P. Ramji , J. Mol. Med. 2002, 80, 753. 12483461 10.1007/s00109-002-0384-9

[5] M. Cuchel , E. Bruckert , H. N. Ginsberg , F. J. Raal , R. D. Santos , R. A. Hegele , J. A. Kuivenhoven , B. G. Nordestgaard , O. S. Descamps , E. Steinhagen‐Thiessen , A. Tybjærg‐Hansen , G. F. Watts , M. Averna , C. Boileau , J. Borén , A. L. Catapano , J. C. Defesche , G. K. Hovingh , S. E. Humphries , P. T. Kovanen , L. Masana , P. Pajukanta , K. G. Parhofer , K. K. Ray , A. F. Stalenhoef , E. Stroes , M. R. Taskinen , A. Wiegman , O. Wiklund , M. J. Chapman , European Atherosclerosis Society Consensus Panel on Familial Hypercholesterolaemia, Turk. Kardiyol. Dern Ars, 2015, 43, 1. 27326442

[6] K. Musunuru , J. P. Pirruccello , R. Do , G. M. Peloso , C. Guiducci , C. Sougnez , K. V. Garimella , S. Fisher , J. Abreu , A. J. Barry , T. Fennell , E. Banks , L. Ambrogio , K. Cibulskis , A. Kernytsky , E. Gonzalez , N. Rudzicz , J. C. Engert , M. A. DePristo , M. J. Daly , J. C. Cohen , H. H. Hobbs , D. Altshuler , G. Schonfeld , S. B. Gabriel , P. Yue , S. Kathiresan , N. Engl. J. Med. 2010, 363, 2220. 20942659 10.1056/NEJMoa1002926 PMC3008575

[7] S. Kersten , Nat. Rev. Endocrinol. 2017, 13, 731. 28984319 10.1038/nrendo.2017.119

[8] Y. Wang , M. C. McNutt , S. Banfi , M. G. Levin , W. L. Holland , V. Gusarova , J. Gromada , J. C. Cohen , H. H. Hobbs , Proc. Natl. Acad. Sci. USA 2015, 112, 11630. 26305978 10.1073/pnas.1515374112 PMC4577179

[9] F. J. Raal , R. S. Rosenson , L. F. Reeskamp , G. K. Hovingh , J. J. P. Kastelein , P. Rubba , S. Ali , P. Banerjee , K.‐C. Chan , D. A. Gipe , N. Khilla , R. Pordy , D. M. Weinreich , G. D. Yancopoulos , Y. Zhang , D. Gaudet , N. Engl. J. Med. 2020, 383, 711. 32813947 10.1056/NEJMoa2004215

[10] A. Wiegman , S. Greber‐Platzer , S. Ali , M. D. Reijman , E. A. Brinton , M. J. Charng , S. Srinivasan , C. Baker‐Smith , S. Baum , J. A. Brothers , J. Hartz , P. M. Moriarty , J. Mendell , S. Bihorel , P. Banerjee , R. T. George , B. Hirshberg , R. Pordy , Circulation 2023. 10.1161/CIRCULATIONAHA.123.065529 PMC10814999 37860863

[11] R. Kaplan , T. Zhang , M. Hernandez , F. X. Gan , S. D. Wright , M. G. Waters , T.‐Q. Cai , J. Lipid Res. 2003, 44, 136. 12518032 10.1194/jlr.m200367-jlr200

[12] J. L. Goldstein , M. S. T. L. D. L. Brown , Arterioscler Thromb. Vasc. Biol. 2009, 29, 431. 19299327 10.1161/ATVBAHA.108.179564 PMC2740366

[13] J. D. Horton , N. A. Shah , J. A. Warrington , N. N. Anderson , S. W. Park , M. S. Brown , J. L. Goldstein , Proc. Natl. Acad. Sci. USA 2003, 100, 12027. 14512514 10.1073/pnas.1534923100 PMC218707

[14] L. Vergnes , R. G. Chin , T. de Aguiar Vallim , L. G. Fong , T. F. Osborne , S. G. Young , K. Reue , J. Lipid Res. 2016, 57, 410. 26685326 10.1194/jlr.M064022 PMC4766990

[15] S. Rong , V. A. Cortés , S. Rashid , N. N. Anderson , J. G. McDonald , G. Liang , Y.‐A. Moon , R. E. Hammer , J. D. Horton , Elife 2017, 6, 25015. 10.7554/eLife.25015 PMC5348127 28244871

[16] C. J. Packard , T. Demant , J. P. Stewart , D. Bedford , M. J. Caslake , G. Schwertfeger , A. Bedynek , J. Shepherd , D. Seidel , J. Lipid Res. 2000, 41, 305. 10681415

[17] M. Raabe , M. M. Véniant , M. A. Sullivan , C. H. Zlot , J. Björkegren , L. B. o. Nielsen , J. S. Wong , R. L. Hamilton , S. G. Young , J. Clin. Invest. 1999, 103, 1287. 10225972 10.1172/JCI6576 PMC408359

[18] D. J. Rader , J. J. Kastelein , Circulation 2014, 129, 1022. 24589695 10.1161/CIRCULATIONAHA.113.001292

[19] I. J. Goldberg , J. Lipid Res. 1996, 37, 693. 8732771

[20] A. Koussounadis , S. P. Langdon , I. H. Um , D. J. Harrison , V. A. Smith , Sci. Rep. 2015, 5, 10775. 26053859 10.1038/srep10775 PMC4459080

[21] M. Ratnadiwakara , M. L. Anko , Bio Protoc. 2018, 8, 3072. 10.21769/BioProtoc.3072 PMC8342049 34532533

[22] L. Pérez de Isla , J. L. Díaz‐Díaz , M. J. Romero , O. Muñiz‐Grijalvo , J. D. Mediavilla , R. Argüeso , J. F. Sánchez Muñoz‐Torrero , P. Rubio , P. Álvarez‐Baños , P. Ponte , D. Mañas , L. Suárez Gutierrez , J. M. Cepeda , M. Casañas , F. Fuentes , C. Guijarro , M. Ángel Barba , A. Saltijeral Cerezo , T. Padró , P. Mata , Circulation 2023, 147, 1436. 37009731 10.1161/CIRCULATIONAHA.122.062557 PMC10158600

[23] J. C. Defesche , S. S. Gidding , M. Harada‐Shiba , R. A. Hegele , R. D. Santos , A. S. Wierzbicki , Nat. Rev. Dis. Primers 2017, 3, 17093. 29219151 10.1038/nrdp.2017.93

[24] Y. Wang , V. Gusarova , S. Banfi , J. Gromada , J. C. Cohen , H. H. Hobbs , J. Lipid Res. 2015, 56, 1296. 25954050 10.1194/jlr.M054882 PMC4479334

[25] Y.‐X. Xu , V. Redon , H. Yu , W. Querbes , J. Pirruccello , A. Liebow , A. Deik , K. Trindade , X. Wang , K. Musunuru , C. B. Clish , C. Cowan , K. Fizgerald , D. Rader , S. Kathiresan , Atherosclerosis 2018, 268, 196. 29183623 10.1016/j.atherosclerosis.2017.08.031 PMC5750127

[26] P. Li , X. Ruan , L. Yang , K. Kiesewetter , Y. Zhao , H. Luo , Y. Chen , M. Gucek , J. Zhu , H. Cao , Cell Metab. 2015, 21, 455. 25738460 10.1016/j.cmet.2015.02.004 PMC4350020

