# Supplementary material for: Depletion of Hepatic SREBP2 Protects Against Hypercholesterolemia and Atherosclerosis through the ANGPTL3‐LPL Axis
Source: Adv Sci (Weinh). 2025 Mar 19;12(18):2412677. doi: 10.1002/advs.202412677 (PMC12079391; doi:10.1002/advs.202412677)
Supplement: Supplementary file 1 — Supporting Information [file ADVS-12-2412677-s002.docx]

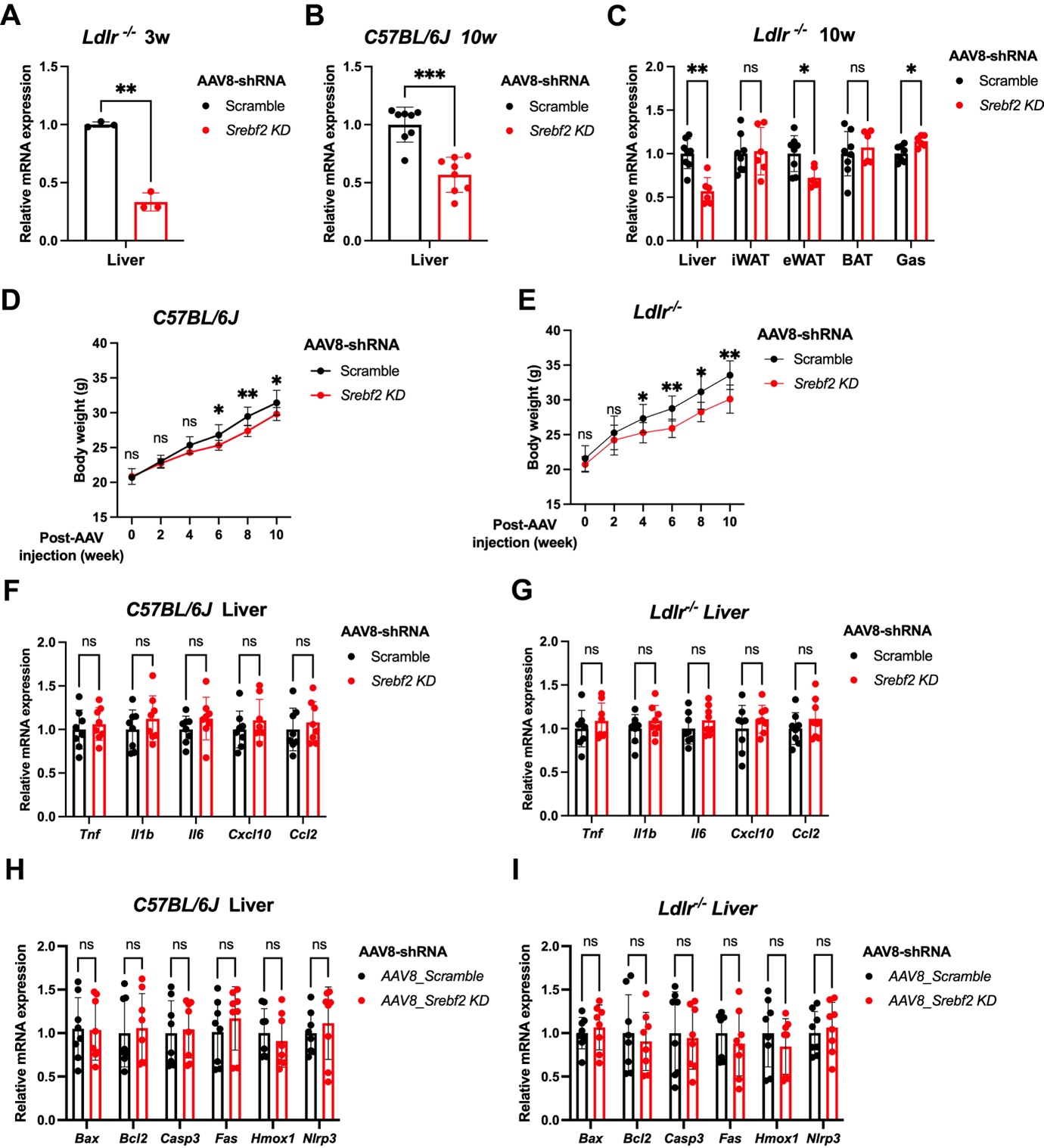


**Figure S1 AAV8-shRNA-*Srebf2* achieves desired knockdown efficiency and specificity in mouse liver.** **Related to Figure 1**

**(A**) Relative expression of *Srebf2* mRNA in the liver of 16-hour fasted male LDLR-deficient mice 3 weeks after injection of AAV8-shRNA-*scramble* or AAV8-shRNA*-Srebf2* viruses, measured via qPCR (n=3 mice per group, by student’s t test).

**(B**) Relative expression of *Srebf2* mRNA in the liver of 16-hour fasted male C57BL/6J mice 10 weeks after injection of AAV8-shRNA-*scramble* or AAV8-shRNA*-Srebf2* viruses, measured via qPCR (n=8 mice per group, by student’s t test).

**(C)** Relative expression of *Srebf2* mRNA in the liver, ingWAT, eWAT, BAT and Gastrocnemius muscle of 16-hour fasted male LDLR-deficient mice 10 weeks after injection of AAV8-shRNA-*scramble* or AAV8-shRNA*-Srebf2* viruses, measured via qPCR (n=6-8 mice per group, by student’s t test).

**(D and E)** Changes in body weight of C57BL/6J (D) and LDLR-deficient (E) male mice injected with AAV8-shRNA-*scramble* or AAV8-shRNA*-Srebf2* viruses under 5-week chow followed by 5 weeks of western diet feeding (n=6-8 mice per group, by two-way ANOVA).

**(F-I)** Relative expression of proinflammatory cytokines or liver injury markers genes in the liver of 16-hour fasted male C57BL/6J (F and H) and *Ldlr^-/-^* (G and I) mice injected with AAV8-scramble control or AAV8-*Srebf2*-shRNA viruses. Mice were maintained on a 5-week chow diet, followed by a 5-week western diet after AAV injection (n=6-8 mice per group, by student’s t test).

* *p*< 0.05, ** *p*< 0.01, *** p<0.001; Error bar indicate mean ± SD.


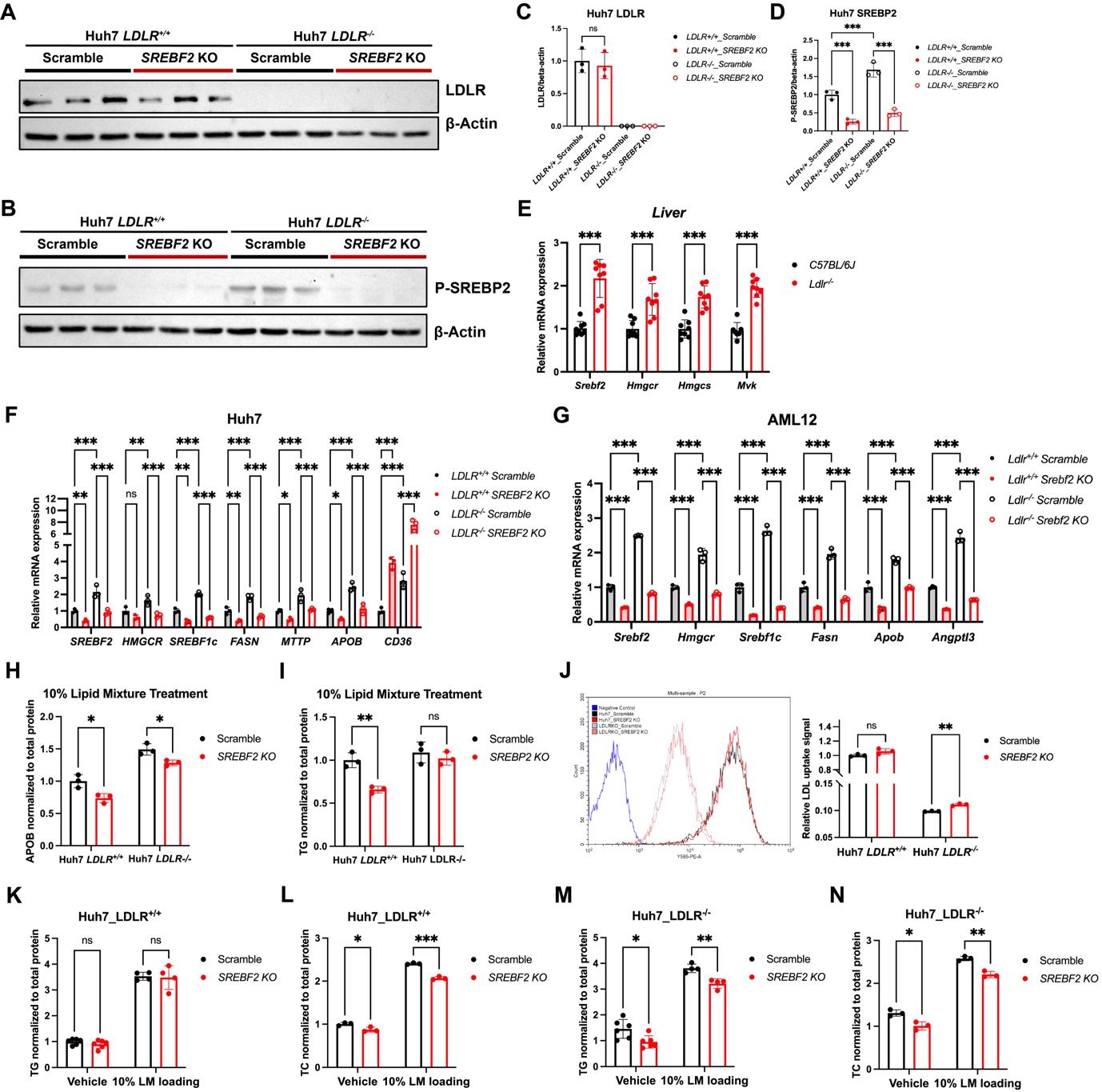


**Figure S2 CRISPR/Cas9-mediated knockout of *SREBF2* reduced cholesterol/ fatty acid biosynthesis and APOB100 secretion *in vitro*. Related to Figure 2**

**(A and C**) Western Blot and quantification of LDLR protein expression in normal and isogenic *LDLR*^-/-^ Huh7 human hepatoma cell lines (n=3 replicates per experiment, representative of 3 independent experiments, by student’s t test).

**(B and D)** Western Blot of precursor SREBP2 protein in *SREBF2* knockout and control *LDLR^+/+^* or *LDLR^-/-^* Huh7 human hepatoma cells (representative of 3 independent experiments, by two-way ANOVA).

(E) Relative mRNA levels of *Srebf2* and its downstream target genes in C57BL/6J and LDLR-deficient male mice injected with AAV8-shRNA-*scramble* for 10 weeks (n=6-8 mice per group, by student’s t test).

**(F)** Relative mRNA levels of *SREBF2* and its downstream genes related to *de novo* lipogenesis and cholesterol biosynthesis in *SREBF2* knockout or control Huh7 cells with or without LDLR. All sample values are normalized relative to *LDLR^+/+^* Scramble (n=3 replicates per experiment, representative of 3 independent experiments, by two-way ANOVA).

**(G)** Relative mRNA levels of *Srebf2* and its downstream genes related to *de novo* lipogenesis and cholesterol biosynthesis in *Srebf2* knockout or control AML12 cells with or without LDLR. All sample values are normalized relative to *Ldlr^+/+^* Scramble (n=3 replicates per experiment, representative of 3 independent experiments, by two-way ANOVA).

**(H and I)** Quantitative analysis of secreted APOB100 (**G**) and TG (**H**) in *SREBF2* knockout or control Huh7 cells with or without LDLR after 10% chemically defined lipid mixture treatment for 24 hours (n=3 replicates per experiment, representative of 3 independent experiments, by two-way ANOVA).

**(J)** Quantification of cellular uptake of fluorophore-labelled LDL particles in *SREBF2* knockout and control Huh7 cells with or without LDLR (n=3 replicates per experiment, representative of 3 independent experiments, by two-way ANOVA).

**(K-N)** Measurement of cellular triglyceride (**J** and **L**) and total cholesterol (**K** and **M**) in *SREBF2* knockout and control Huh7 cells with or without LDLR, in the presence or absence of 10% chemically defined lipid mixture treatment for 24 hours (n=3 replicates per experiment, representative of 3 independent experiments, by two-way ANOVA).

* *p*< 0.05, ** *p*< 0.01, *** p<0.001; Error bar indicate mean ± SD.


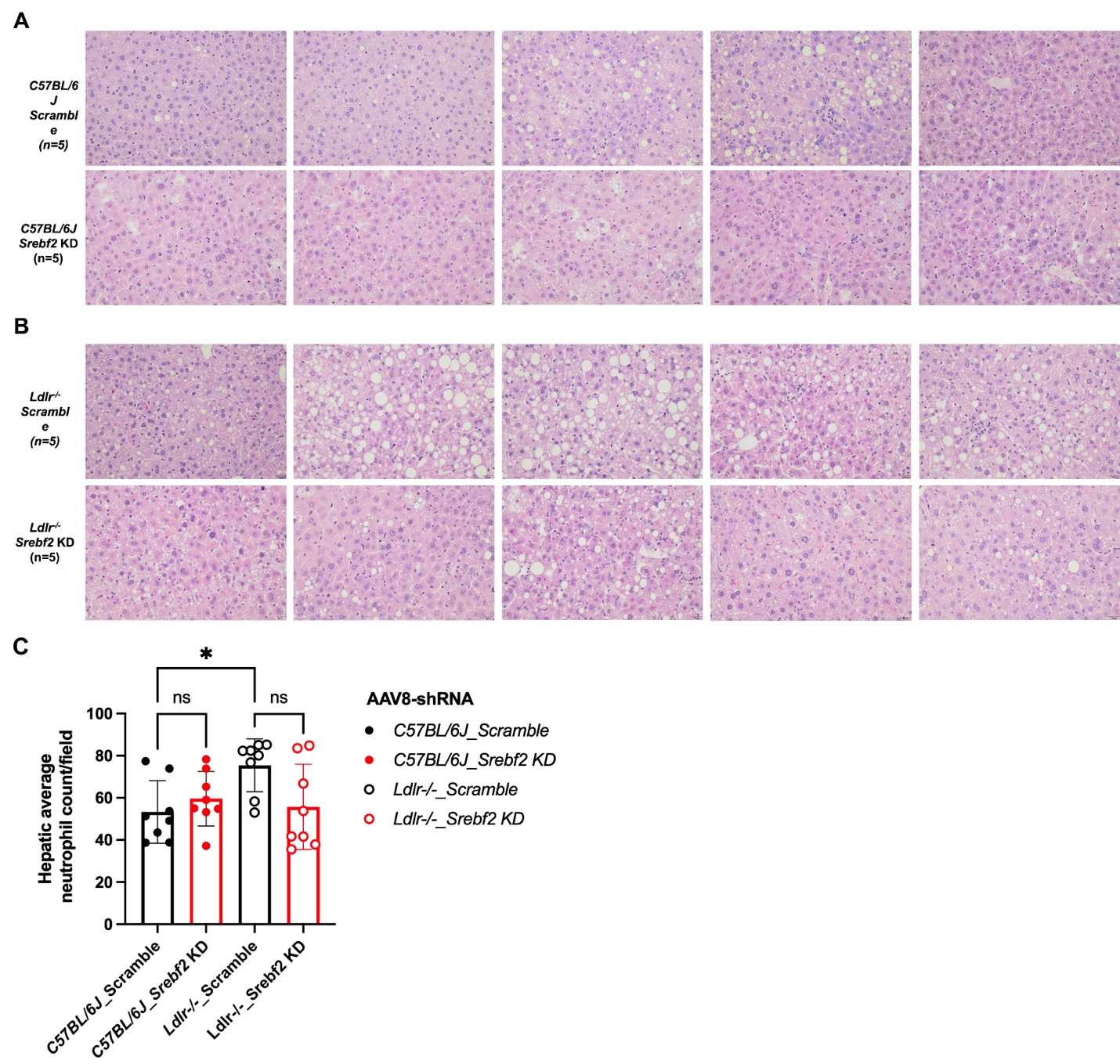


**Figure S3. Acute depletion of hepatic SREBP2 does not exacerbate hepatic steatosis**

**(A-B)** Images of liver sections stained with Hematoxylin & Eosin from male C57BL6/J (A) or LDLR-deficient mice (B) injected with AAV8-shRNA-*scramble* or AAV8-shRNA*-Srebf2* viruses and fed western diet for 14 weeks.

**(C)** Histopathological assessment of average infiltrated neutrophil count/field in liver (n=5, by two-way ANOVA).

* *p*< 0.05, ** *p*< 0.01, *** p<0.001; Error bar indicate mean ± SD.


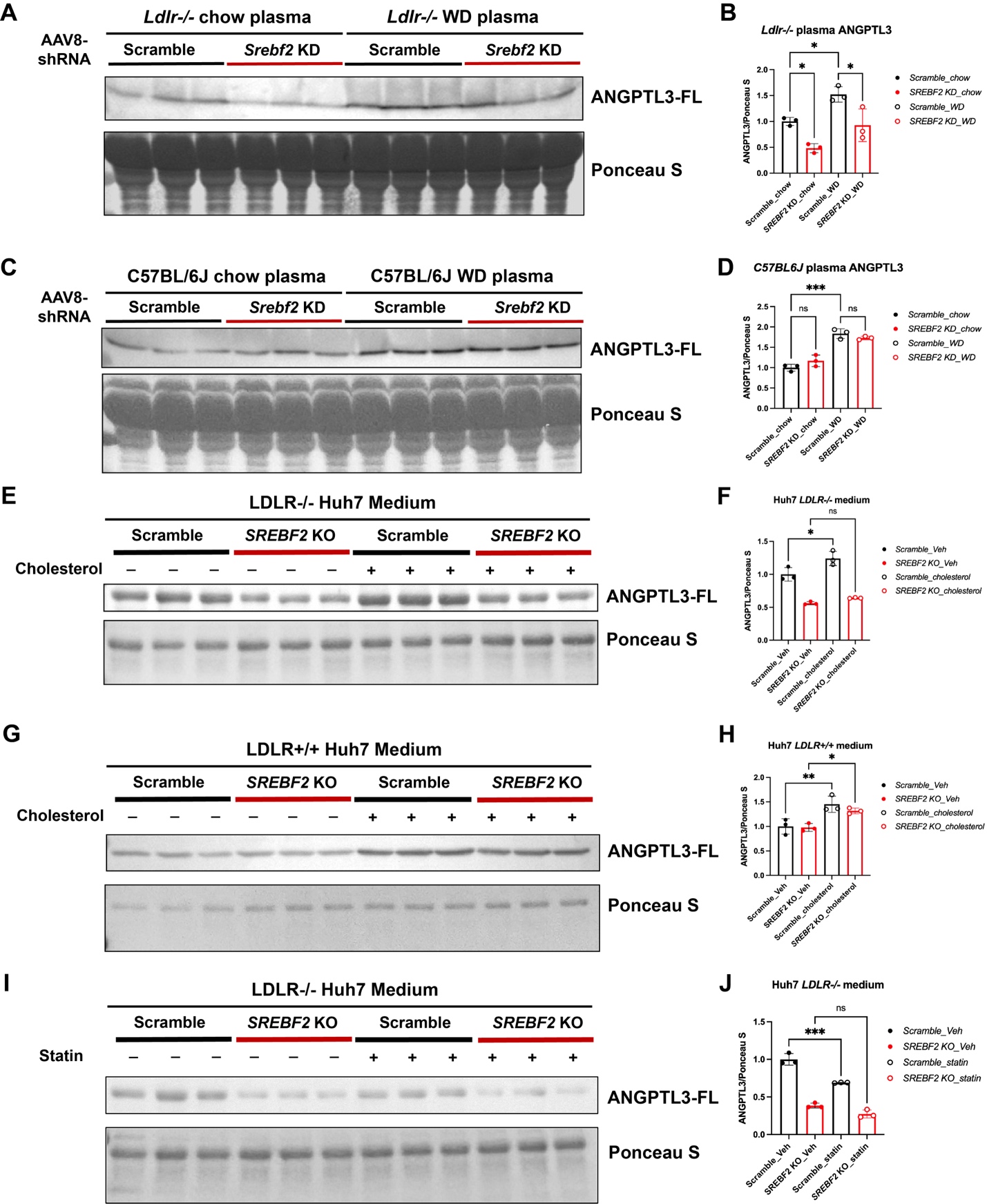


**Figure S4. High cholesterol feeding increases ANGPTL3 secretion independent of SREBP2**

**(A-D)** Western Blot and relative quantification of plasma ANGPTL3 in the heparinized male *Ldlr-/-* mice (A and B) and C57BL/6J (C and D) injected with AAV8-shRNA-*scramble* or AAV8-shRNA*-Srebf2* viruses under chow diet feeding or western diet (WD) feeding. (3 representative mice of each group consisting of 6-8 mice, by two-way ANOVA)

**(E-H)** Western Blot and relative quantification of secreted ANGPTL3 levels in CRISPR/Cas9-mediated *SREBF2* knockout or control *LDLR^-/-^* (E and F) and *LDLR^+/+^* (G and H) Huh7 human hepatoma cells with or without 50uM water-soluble cholesterol treatment for 16 hours. (n=3 replicates from individual wells, representative of 3 independent experiments, by two-way ANOVA).

**(I-J)** Western Blot and relative quantification of secreted ANGPTL3 protein levels in *SREBF2* knockout or control *LDLR^-/-^* Huh7 human hepatoma cells treated with 10uM statin or DMSO (Vehicle) for 16 hours. (n=3 replicates from individual wells, representative of 3 independent experiments, by two-way ANOVA).

* *p*< 0.05, ** *p*< 0.01, *** p<0.001; Error bar indicate mean ± SD.


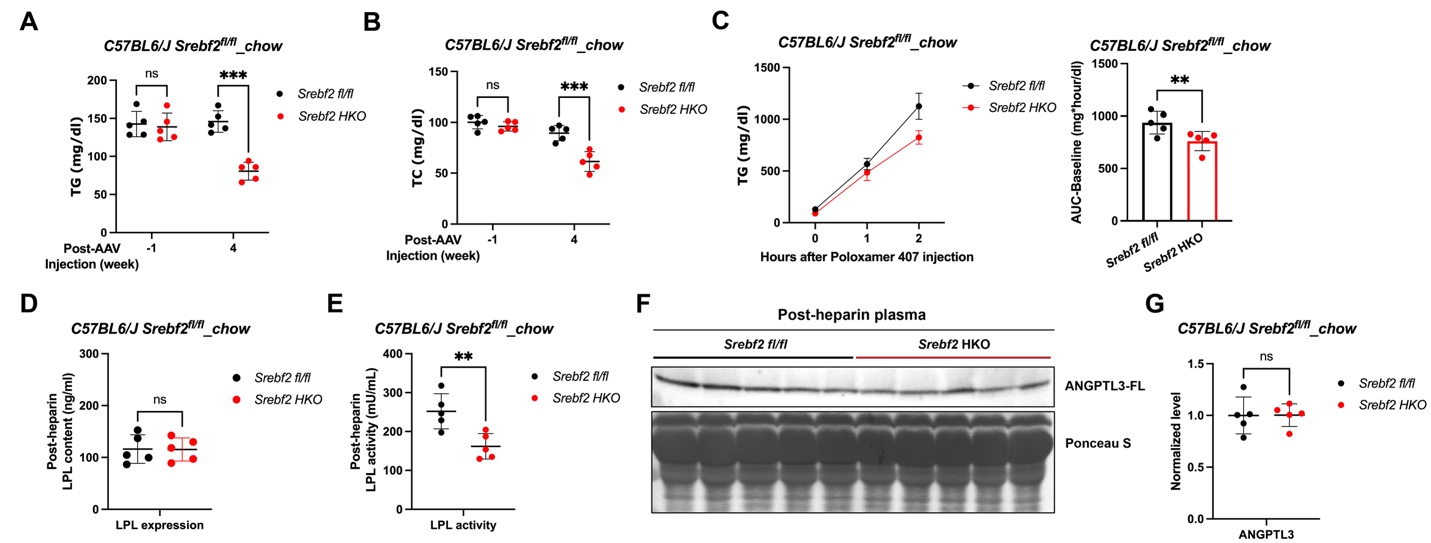


**Figure S5. Hepatocyte-specific depletion of SREBP2 reduces circulating lipids**

**(A-B)** Plasma triglyceride (A) and cholesterol (B) levels of 16h-fasted *Srebf2*^fl/fl^ male mice injected with AAV8-TBG-EGFP or AAV8-TBG-Cre viruses under chow diet feeding (A and B, n=5 mice per group, by two-way ANOVA).

**(C)** Changes of plasma triglyceride after Poloxamer-407 injection in *Srebf2*^fl/fl^ male mice injected with AAV8-TBG-EGFP or AAV8-TBG-Cre viruses under chow diet feeding. VLDL secretion levels were estimated and compared using an area-under-the-curve (AUC) approach that subtracts the baseline value (n=5 mice per group, by student’s t-test).

**(D and E)** Plasma total lipoprotein lipase contents (D) and lipoprotein lipase activity (E) measured in heparinized *Srebf2*^fl/fl^ male mice injected with AAV8-TBG-EGFP or AAV8-TBG-Cre viruses under chow diet feeding (n=5 mice per group, by student’s t test).

**(F and G)** Western blot (F) and relative quantification (G) of plasma ANGPTL3 in heparinized *Srebf2* fl/fl male mice injected with AAV8-TBG-EGFP or AAV8-TBG-Cre viruses (n=5 mice per group, by student’s t test).

* *p*< 0.05, ** *p*< 0.01, *** p<0.001; Error bar indicate mean ± SD.


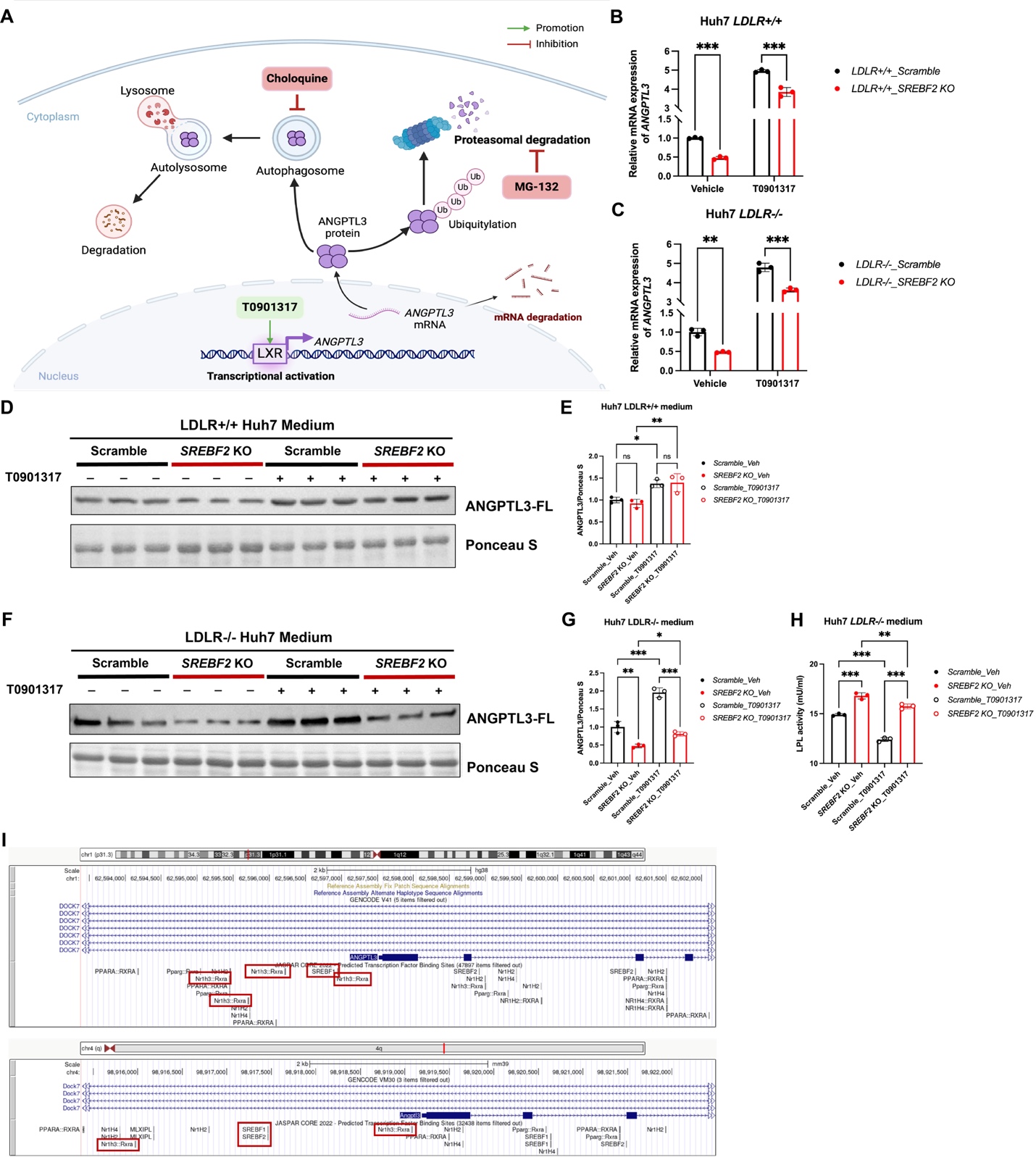


**Figure S6. LXR activation is unable to restore ANGPTL3 expression following depletion of SREBP2**

1. Schematic diagram of transcriptional and post-transcriptional regulation of ANGPTL3.

(B-C) Relative mRNA levels of *ANGPTL3* in *SREBF2* knockout or control Huh7 *LDLR+/+* (B) or *LDLR-/-* (C) cells treated with 1uM T0901317 (LXR agonist) or DMSO (Vehicle) for 16 hours. (n=3 replicates per experiment, representative of 3 independent experiments, by two-way ANOVA).

(D-G) Western blots and relative quantification of secreted ANGPTL3 protein levels in *SREBF2* knockout or control d *LDLR+/+* (D and E) or LDLR-/- (F and G) Huh7 human hepatoma cells treated with 1uM T0901317 (LXR agonist) or DMSO (Vehicle) for 16 hours. (n=3 replicates from individual wells, representative of 3 independent experiments, by two-way ANOVA).

(H) Activity of LPL pretreated with cell culture medium isolated from CRISPR/Cas9-mediated *SREBF2* knockout or control *LDLR^-/-^* Huh7 cells treated with 1uM T0901317 (LXR agonist) or DMSO (Vehicle) for 16 hours. (n=3 replicates from individual wells, representative of 3 independent experiments, by two-way ANOVA).

(I) Transcriptional factor binding site prediction in the promotor region of Human *ANGPTL3* and mouse *Angptl3* using JASPAR database incorporated in UCSC genome browser.

* *p*< 0.05, ** *p*< 0.01, *** p<0.001; Error bar indicate mean ± SD.


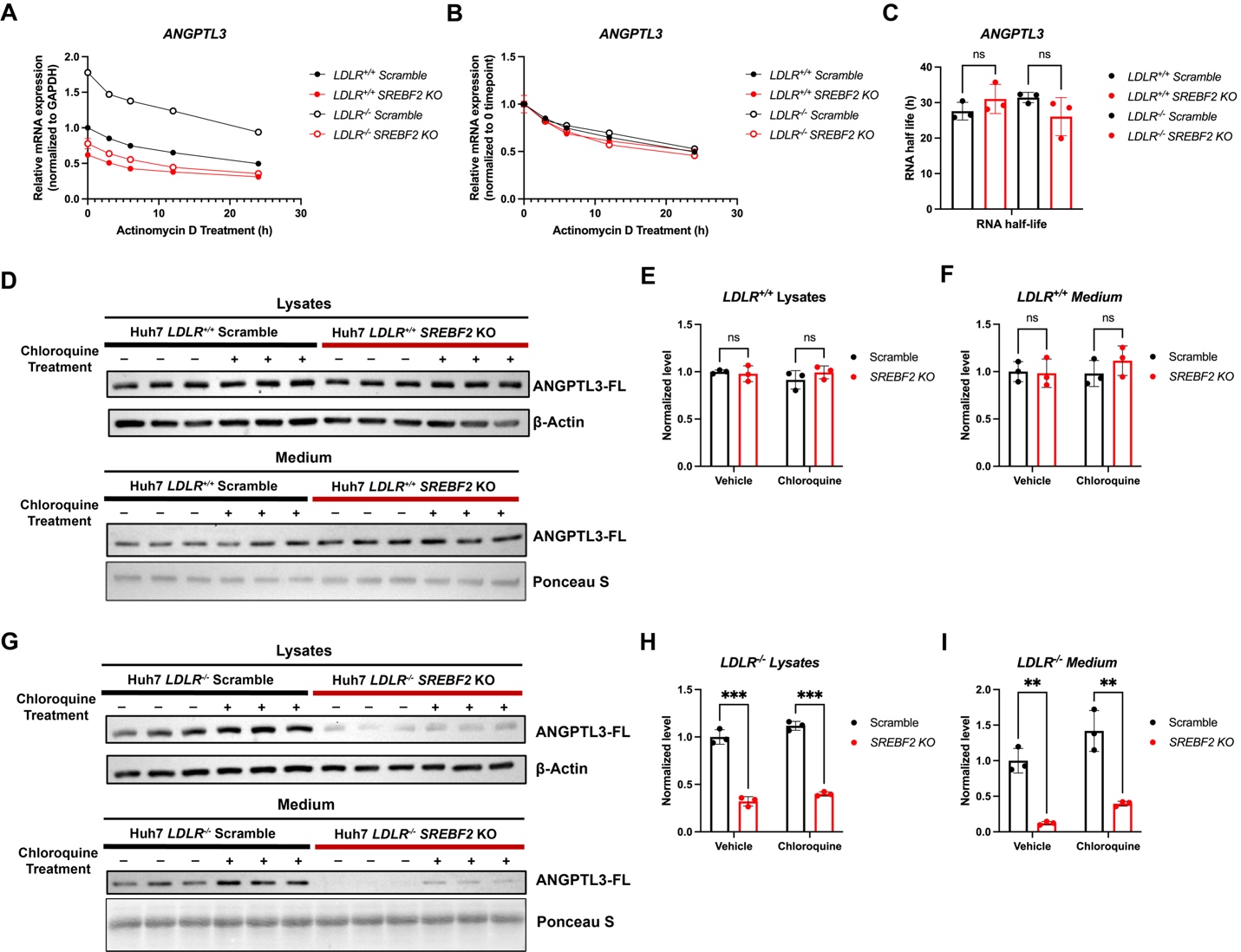


**Figure S7. *ANGPTL3* mRNA stability and lysosomal degradation were not affected by depleting SREBP2.**

**(A-C)** *ANGPTL3* mRNA stability assay performed by treating cells with Actinomycin-D to block transcription and measuring remaining mRNA at 0, 3, 6, 12, 24 hours using different Huh7 hepatoma cell lines. mRNA expression was normalized to GAPDH, a stable transcript (A) or the 0h expression level of each cell line (B). The *ANGPTL3* mRNA half-lives in different Huh7 hepatoma cells were indicated in bar plots (C). (n=3 replicates from individual wells, representative of 3 independent experiments, by two-way ANOVA).

**(D-I)** Western Blot and relative quantification of cellular and secreted ANGPTL3 protein levels in CRISPR/Cas9-mediated *SREBF2* knockout or control *LDLR^+/+^* (D-F) and *LDLR^-/-^* (G-I) Huh7 human hepatoma cells upon 1uM MG-132 or DMSO treatment for 4 hours. (n=3 replicates from individual wells, representative of 3 independent experiments, by two-way ANOVA).

* *p*< 0.05, ** *p*< 0.01, *** p<0.001; Error bar indicate mean ± SD.


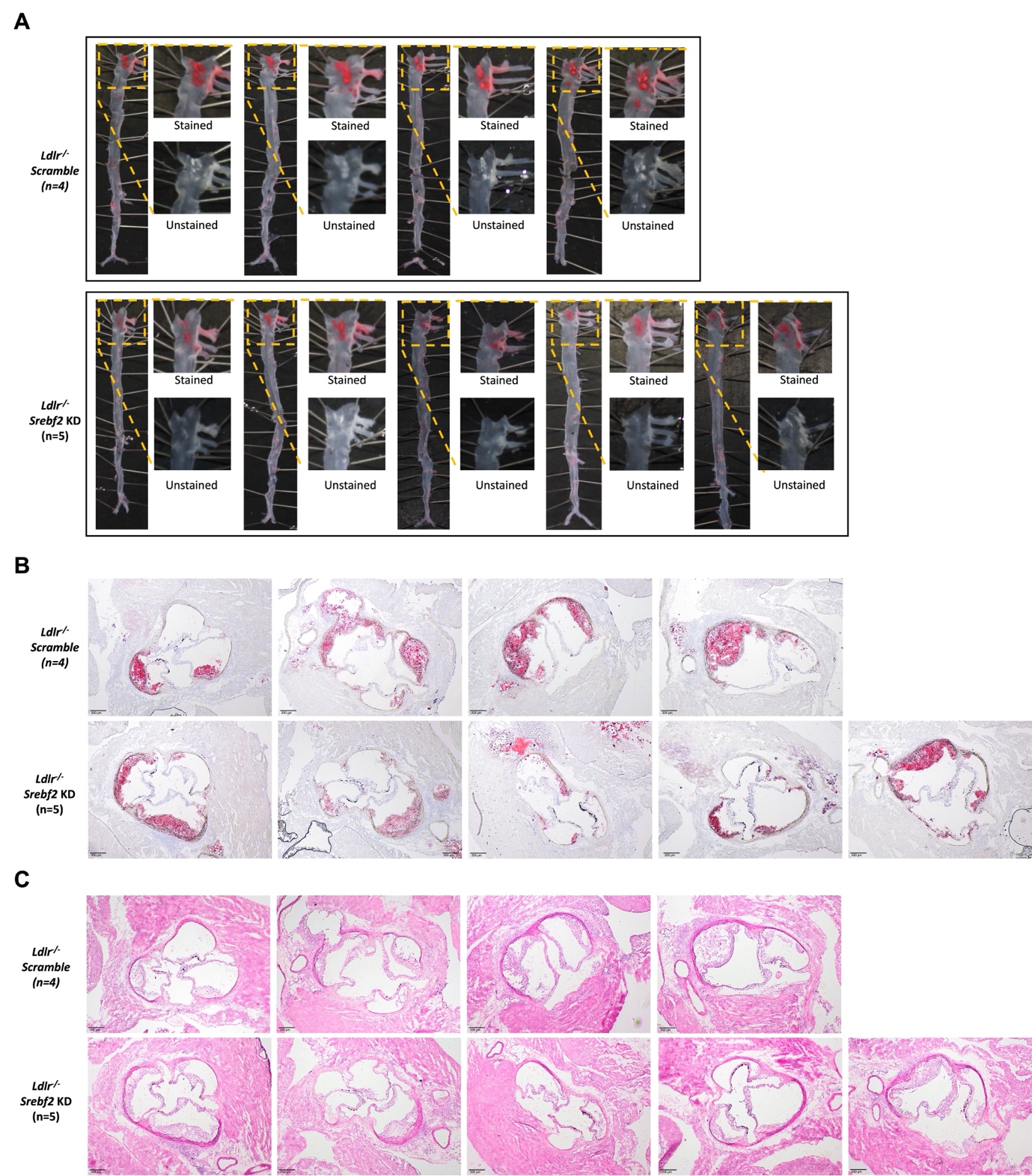


**Figure S8 Acute depletion of hepatic SREBP2 protects against atherosclerosis.**

**(A)** Images of Aortas before and after Oil Red O staining in male LDLR-deficient mice injected with AAV8-shRNA-*scramble* or AAV8-shRNA-*Srebf2* viruses and fed western diet for 14 weeks.

**(B and C)** Images of cross-sectional aortic root sections stained with Oil Red O & Hematoxylin (B) and Hematoxylin & Eosin (C) for aortic lesion areas from male LDLR-deficient mice injected with AAV8-shRNA-*scramble* or AAV8-shRNA-*Srebf2* viruses and fed western diet for 14 weeks.
